# Root exudate composition reflects drought severity gradient in blue grama (*Bouteloua gracilis*)

**DOI:** 10.1038/s41598-022-16408-8

**Published:** 2022-07-22

**Authors:** Danielle E. M. Ulrich, Chaevien S. Clendinen, Franklin Alongi, Rebecca C. Mueller, Rosalie K. Chu, Jason Toyoda, La Verne Gallegos-Graves, Hannah M. Goemann, Brent Peyton, Sanna Sevanto, John Dunbar

**Affiliations:** 1 Ecology Department, Montana State University, Bozeman, MT USA; 2 Environmental Molecular Sciences Laboratory, Pacific Northwest National Laboratory, Richland, WA USA; 3 Plant Science and Plant Pathology Department, Montana State University, Bozeman, MT USA; 4 Western Regional Research Center, Agricultural Research Service, Albany, CA USA; 5 Bioscience Division, Los Alamos National Laboratory, Los Alamos, NM USA; 6 Microbiology and Cell Biology Department, Montana State University, Bozeman, MT USA; 7 Thermal Biology Institute, Montana State University, Bozeman, MT USA; 8 Earth and Environmental Sciences Division, Los Alamos National Laboratory, Los Alamos, NM USA

**Keywords:** Drought, Plant physiology, Ecophysiology, Metabolomics

## Abstract

Plant survival during environmental stress greatly affects ecosystem carbon (C) cycling, and plant–microbe interactions are central to plant stress survival. The release of C-rich root exudates is a key mechanism plants use to manage their microbiome, attracting beneficial microbes and/or suppressing harmful microbes to help plants withstand environmental stress. However, a critical knowledge gap is how plants alter root exudate concentration and composition under varying stress levels. In a greenhouse study, we imposed three drought treatments (control, mild, severe) on blue grama (*Bouteloua gracilis* Kunth Lag. Ex Griffiths), and measured plant physiology and root exudate concentration and composition using GC–MS, NMR, and FTICR. With increasing drought severity, root exudate total C and organic C increased concurrently with declining predawn leaf water potential and photosynthesis. Root exudate composition mirrored the physiological gradient of drought severity treatments. Specific compounds that are known to alter plant drought responses and the rhizosphere microbiome mirrored the drought severity-induced root exudate compositional gradient. Despite reducing C uptake, these plants actively invested C to root exudates with increasing drought severity. Patterns of plant physiology and root exudate concentration and composition co-varied along a gradient of drought severity.

## Introduction

Drought conditions are increasing in frequency and severity, challenging our ability to understand and predict terrestrial ecosystem functions such as carbon (C) and nutrient cycling under future climates^1,2^. Plants allocate the C products of photosynthesis to essential functions: growth, reproduction, metabolism, storage, and stress resistance^3^. An understudied component of plant C allocation is C released outside the plant and to the soil as root exudates^4^. Root exudates can alter ecosystem functions including C cycling via changing soil characteristics, increasing nutrient availability, and stimulating the rhizosphere microbial community^5–7^. However, how droughts of varying severity affect root exudates, plant–microbe interactions, and C and nutrient cycling remains poorly understood^8^.

Root exudates are C-rich compounds including sugars, amino acids, organic acids, phenolics, secondary metabolites, proteins, and lipids that can influence ecosystem function under drought through multiple pathways^9^. First, root exudates can alter ecosystem C cycling by stabilizing soil organic C (via biochemical recalcitrance, mineral adsorption, physical inaccessibility) or destabilizing soil organic C (via stimulating microbial activity and decomposition)^7^. Second, root exudates can alter soil properties by increasing soil moisture holding capacity^10^ and changing physical soil structure^11^. These changes can delay the impacts of drought on plant function^12^ and improve the ability of roots to access deeper water sources^13^. Third, root exudates can enhance the availability of soil nutrients for both plants and microorganisms (e.g. nitrogen, carbon) directly by the addition of nutrient-rich compounds and indirectly by altering nutrient availability. For example, organic acids of root exudates can break bonds between organic substances and nutrients, increasing plant nutrient availability^14,15^. Organic acids in root exudates can also liberate organic matter from minerals and increase organic matter availability to microbes^16^. Lastly, due to differences in substrate preference among microbial taxa, root exudates can directly shape the taxonomic and functional composition of the rhizosphere microbial community by attracting or repelling certain microbes^17,18^, which in turn may improve plant response to drought^19–21^. For example, root exudates can increase nutrient availability through rhizosphere priming, which stimulates microbial activity including mineralization of soil organic matter^22,23^. Microbes also can directly enhance plant drought resistance strategies by increasing stress signaling (e.g. sugars, amino acids) and producing phytohormones to stimulate root growth for water uptake to sustain photosynthesis^24,25^.

Given the diversity of root exudate compound types and functions, root exudate quantity and composition can greatly affect ecosystem C cycling, soil characteristics, nutrient availability, and the rhizosphere microbial community^6,26^. Therefore, a step towards improving our understanding of ecosystem function and plant–microbe interactions under changing environmental conditions is examining how varying levels of drought severity can influence root exudate quantity and composition. In particular, whether the amount of root exudation increases with drought severity remains unclear^11,27^. In response to drought, some species increase root exudate concentrations^28,29^ while others decrease exudate concentrations^11,30^. The mixed effects of drought severity on root exudate concentration may arise due to variation in a plant species’ use of specific types of compounds for positive (attractive) versus negative (suppressive) manipulation of the rhizosphere microbiome to promote essential plant functions during stress. Therefore, root exudate composition has been observed to differ between droughted, control, and recovering plants^11,31^. This suggests that different drought severity levels may also induce different shifts in root exudate concentration and composition, which may be linked to stress-induced shifts in C allocation and physiology. This linkage between root exudates, physiology, and C allocation exists because drought affects plant C uptake (C assimilation, photosynthesis) and C allocation to above- and belowground pools (roots, shoots) including root exudates^4,32–35^. A severe drought in duration and/or intensity is expected to have a greater effect on C assimilation and/or C allocation shifts than a mild drought, while an intermediate level of drought would yield some intermediate response^36^. As a result, the amount of C allocated to root exudates may also be affected in a similar gradiential pattern (either an increase or decrease) in response to increasing drought severity. Additionally, root exudate compounds that underlie the increase or decrease in root exudate quantity would also exhibit a similar gradiential pattern (either an increase or decrease) in response to increasing drought severity. However, drought severity-induced shifts in plant C allocation root exudate quantity and quality are poorly understood because collecting and analyzing root exudates is difficult and the mechanisms of root exudation remain unclear^20,37,38^. This knowledge gap complicates assessment of C cycling impacts under changing climates.

Blue grama (*Bouteloua gracilis* Kunth Lag. Ex Griffiths) is a widespread, warm-season, perennial, C4 grass that dominates shortgrass steppe communities in North America and accounts for 75–90% of net primary productivity on most sites it inhabits^39^. Such grassland ecosystems are expected to become increasingly warmer and drier^40–42^. Grassland ecosystems are also major C sinks; grassland species can allocate as much as 50% of their C belowground in root biomass and root exudates^43^. However, the potential for grasslands to store C under climate change will increasingly depend upon precipitation regimes because grassland productivity is even more responsive to precipitation pulses than productivity of forest ecosystems^44,45^. Blue grama’s relationship with soil microbes may contribute to its drought resistance, nutrient acquisition, and wide geographic distribution^46^. For example, blue grama rhizosphere soils exhibited greater soluble sugars and polyphenols than non-rhizosphere soil^47,48^. Together, this suggests that drought influences blue grama root exudate concentration and composition. In spite of this, the effects of varying drought severity on blue grama root exudate concentration and composition remain unexplored. Given that ~ 46% of Poaceae (grass family) are C4^49^, that blue grama can adapt to diverse environmental conditions^50–52^, and that warm season perennial C4 grasses have been observed to respond similarly to stress^53,54^, investigating blue grama physiology and C allocation informs other warm-season perennial C4 grass species’ responses to stress.

This study investigated the influence of drought severity on blue grama physiology and root exudate concentration and composition. We imposed three drought severity treatments (control, mild, severe) on blue grama plants grown in pots from seed, and measured plant physiological traits (predawn leaf water potential, photosynthesis, stomatal conductance, root to shoot biomass ratio (root:shoot), total biomass), root exudate C concentration (total C, organic C, inorganic C), and root exudate composition using three metabolomic platforms. We hypothesized that: (1) blue grama physiology, the concentration of root exudate C, and the quantity of specific root exudate compounds would change (increase or decrease) concurrently with increasing drought severity, and (2) root exudate compounds, that may alter plant drought resistance and/or the rhizosphere microbiome, would increase concurrently with increasing drought severity.

## Results

### Plant physiology and root exudate C reflected the drought severity gradient

**Figure 1:**
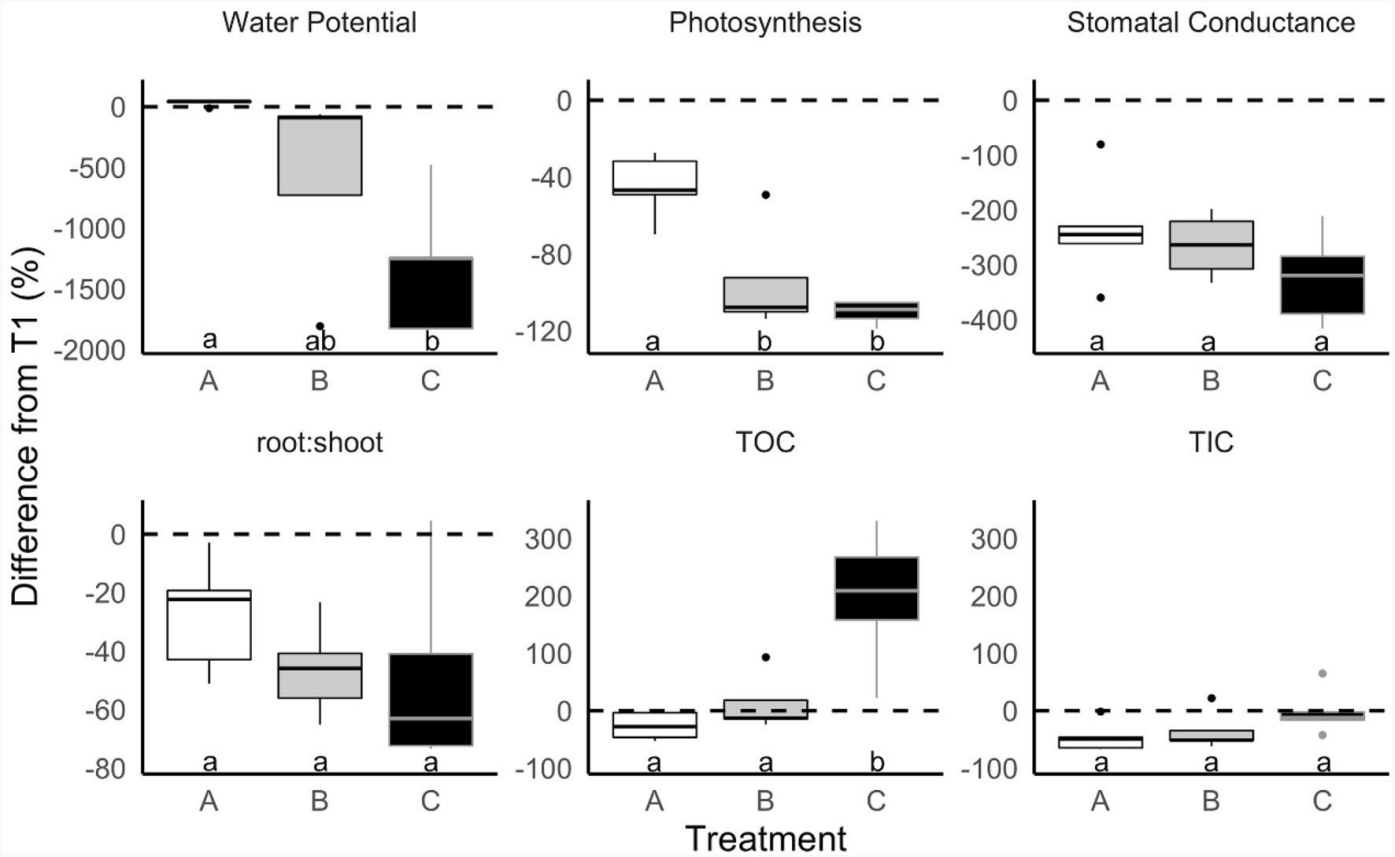
Drought severity treatments (A = conrol, B = mild, C = severe) affected plant physiology and root exudate concentration concurrently. The percent difference between before (T1) and after treatment (T2) on four physiological metrics: predawn leaf water potential, photosynthesis, stomatal conductance, growth (root:shoot biomass), and two metrics of root exudate concentration: total organic C (TOC) and root exudate total inorganic C (TIC). Lowercase letters indicate significant differences in means among treatments at T2 (*p* < 0.05, Table 1, Supplementary Table S1). Note the different y-axis scales.

At T2, all physiological and root exudate quantity measurements exhibited the same trend of an increasing magnitude of response from treatment A (control) to B (mild) to C (severe), reflecting the drought severity treatment gradient (Fig. 1, Table 1). The percent difference between T1 and T2 for mean predawn leaf water potential, photosynthesis, stomatal conductance, root:shoot, and total biomass increasingly declined with increasing drought severity, with treatment C exhibiting the greatest declines in these variables with B showing an intermediate decline and A showing the smallest decline (Fig. 1, Supplementary Tables S1, S2). Root exudate TC and TOC exhibited the same trend but in the opposite direction, where the percent difference between T1 and T2 for TC and TOC increased with increasing drought severity (Fig. 1, Supplementary Table S1). As expected, this pattern of physiology and root exudate C content shifting with increasing drought severity was not observed at T1. None of the physiological or root exudate measurements significantly differed among treatments at T1 (*p* > 0.05, Table 1). At T2, mean predawn leaf water potential, photosynthesis, TC, and TOC significantly differed between treatments (*p* < 0.05, Fig. 1, Table 1, Supplementary Tables S1, S2), while mean stomatal conductance, root:shoot, and TIC did not (*p* > 0.05, Fig. 1, Table 1, Supplementary Tables S1, S2). The significant treatment difference in TC was driven by TOC because TIC did not differ significantly among treatments (Table 1). Mean values of physiological and root exudate quantity measurements are reported in Supplementary Table S2.

**Table 1:** ANOVA tables identifying significant differences between treatment means before (T1) and after treatment (T2) for each physiological response variable (predawn leaf water potential, photosynthesis, stomatal conductance, root to shoot biomass ratio (root:shoot), root exudate total C (TC), root exudate total organic C (TOC), root exudate total inorganic C (TIC), total (root + shoot) biomass).

	T1					T2				
	DF	SS	MS	F	*p* Value	DF	SS	MS	F	*p* Value
**Predawn leaf water potential**										
Treatment	2	2.46	1.23	0.35	0.71	2	12,532.20	6266.10	7.92	** < 0.01**
Residuals	12	41.62	3.47			12	9494.50	791.20		
**Photosynthesis**										
Treatment	2	0.19	0.09	0.02	0.98	2	13.16	6.58	15.65	** < 0.001**
Residuals	12	73.67	6.14			11	4.63	0.42		
**Stomatal conductance**										
Treatment	2	0.00	0.00	1.24	0.32	2	0.00	0.00	1.42	0.28
Residuals	12	0.00	0.00			11	0.00	0.00		
**Root:shoot**										
Treatment	2	0.94	0.47	1.65	0.23	2	0.55	0.28	1.65	0.23
Residuals	12	3.41	0.28			12	2.01	0.17		
**Total (root + shoot) biomass**										
Treatment	2	67,637	33,818	1.55	0.25	2	61,070	30,535	4.26	**0.040**
Residuals	12	261,665	21,805			12	85,918	7160		
**TC**										
Treatment	2	2.04	1.02	3.88	0.05	2	2.65	1.32	10.13	** < 0.01**
Residuals	12	3.15	0.26			12	1.57	0.13		
**TOC**										
Treatment	2	1.64	0.82	3.81	0.05	2	4.83	2.42	14.45	** < 0.001**
Residuals	12	2.58	0.22			12	2.01	0.17		
**TIC**										
Treatment	2	2.28	1.14	3.59	0.06	2	1.01	0.51	2.84	0.10
Residuals	12	12.00	3.81	0.32		12	2.13	0.18		

*DF* degrees of freedom, *SS* sum of squares, *MS* mean square, *F* F-statistic, *p* value. Bold values indicate *p *< 0.05.

### Root exudate composition reflected the drought severity gradient

**Figure 2:**
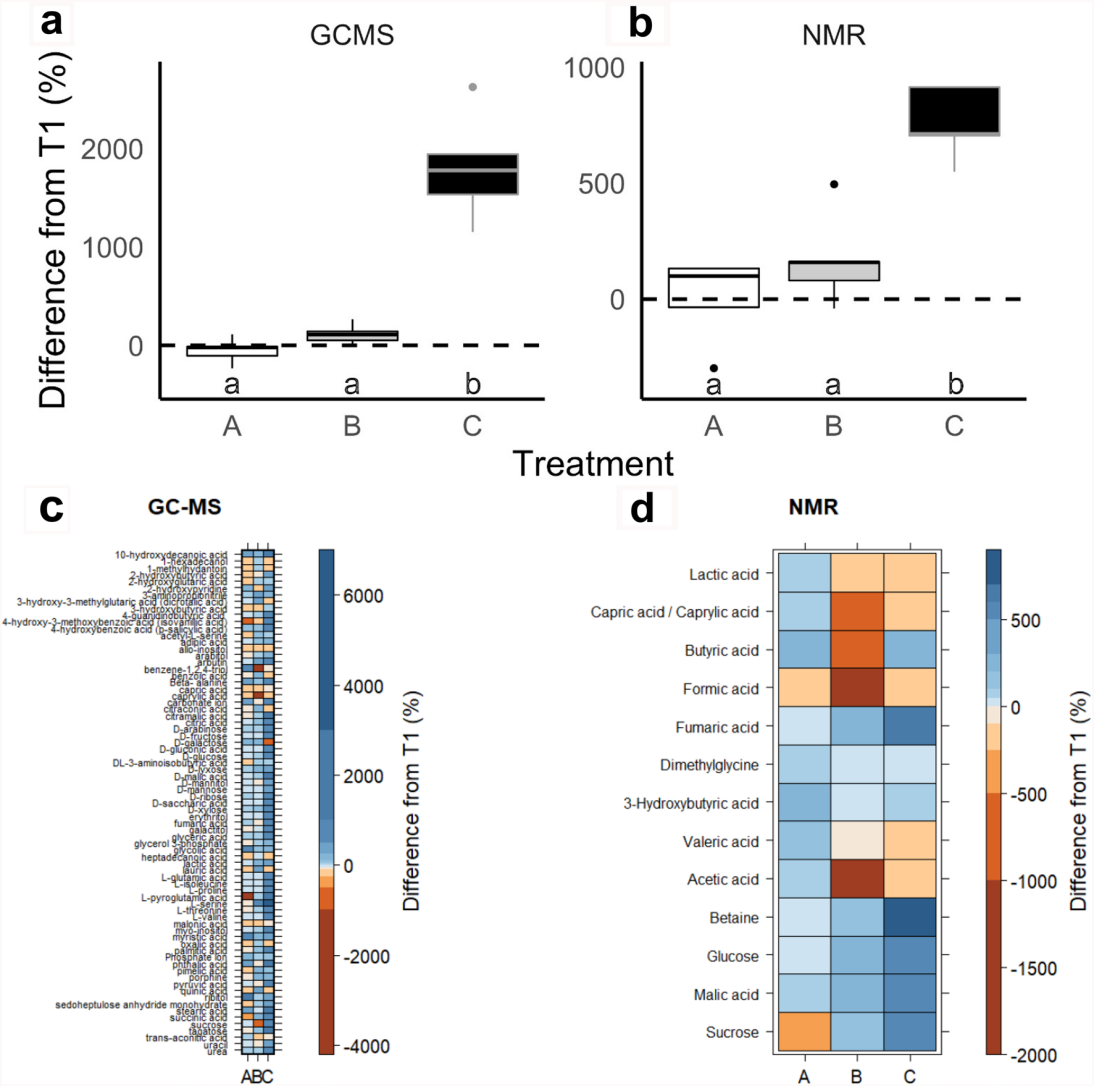
Drought severity treatments (A = conrol, B = mild, C = severe) affected normalized peak area of GC–MS and NMR concurrently. The percent difference between before (T1) and after treatment (T2) of normalized peak area of GC–MS (**a**) and NMR (**b**). Lowercase letters indicate significant differences in means among treatments at T2 (*p* < 0.05, Supplementary Tables S3, S4). Note the different y-axis scales. Heat maps describing the percent difference between T1 and T2 of z-scores of identified metabolites for each treatment identified with GC–MS (**c**) and NMR (**d**). List of GC–MS compounds in panel C in Supplementary Table S5. 64 of 206 GC–MS detected metabolites (32 of the 64 were identified) increased from A to B to C and 28 of 206 GC–MS detected metabolites (8 of the 28 were identified) decreased from A to B to C. 5 of 13 NMR detected metabolites increased from A to B to C and 3 of 13 NMR detected metabolites decreased from A to B to C.

**Figure 3:**
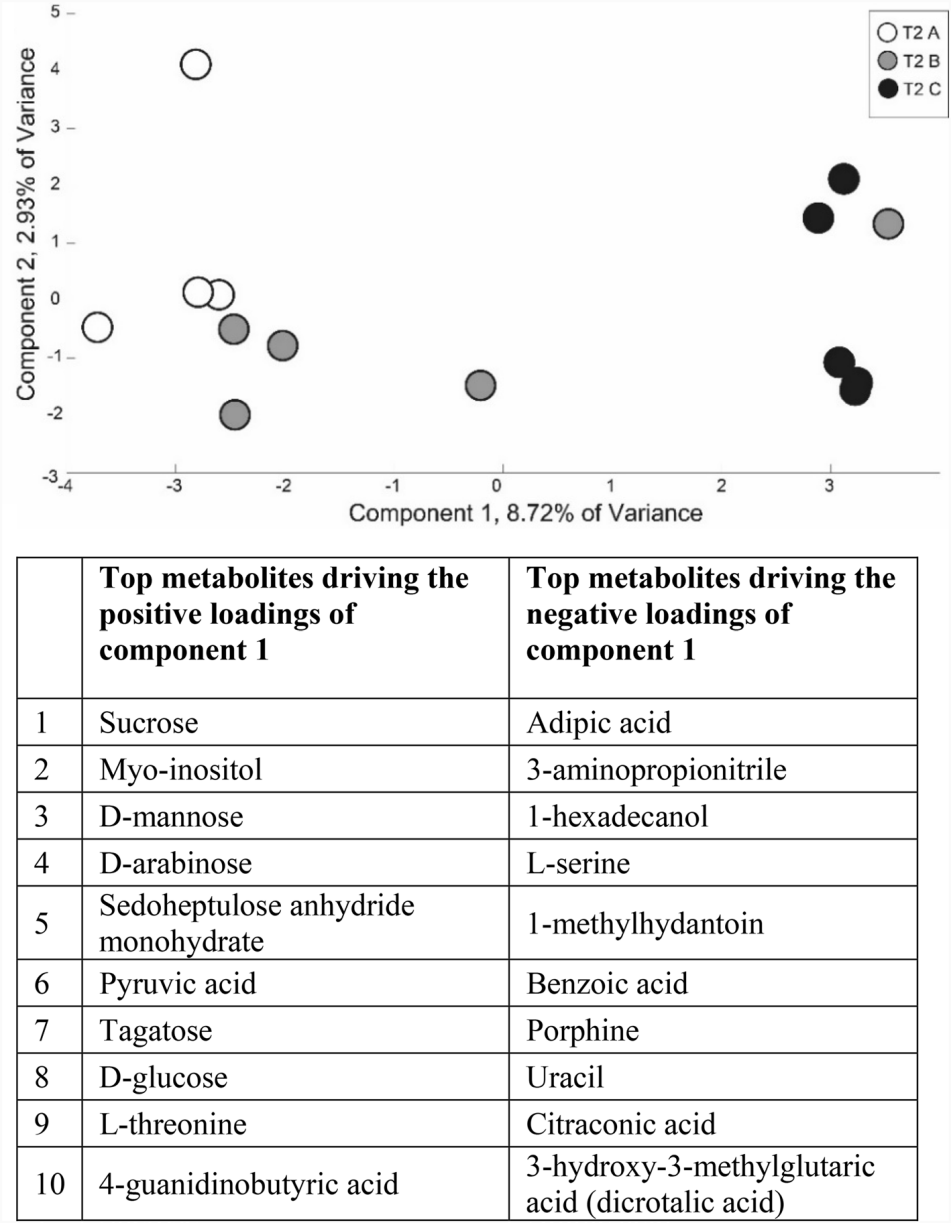
GC–MS-identified root exudate compounds associated with driving the treatment separation, reflecting the drought severity treatment gradient. GC–MS probabilistic PCA (pPCA) loadings and scores of treatments A (control), B (mild), and C (severe) after treatment (T2). Table lists the top ten metabolites driving the positive and negative pPCA loadings of component 1.

**Figure 4:**
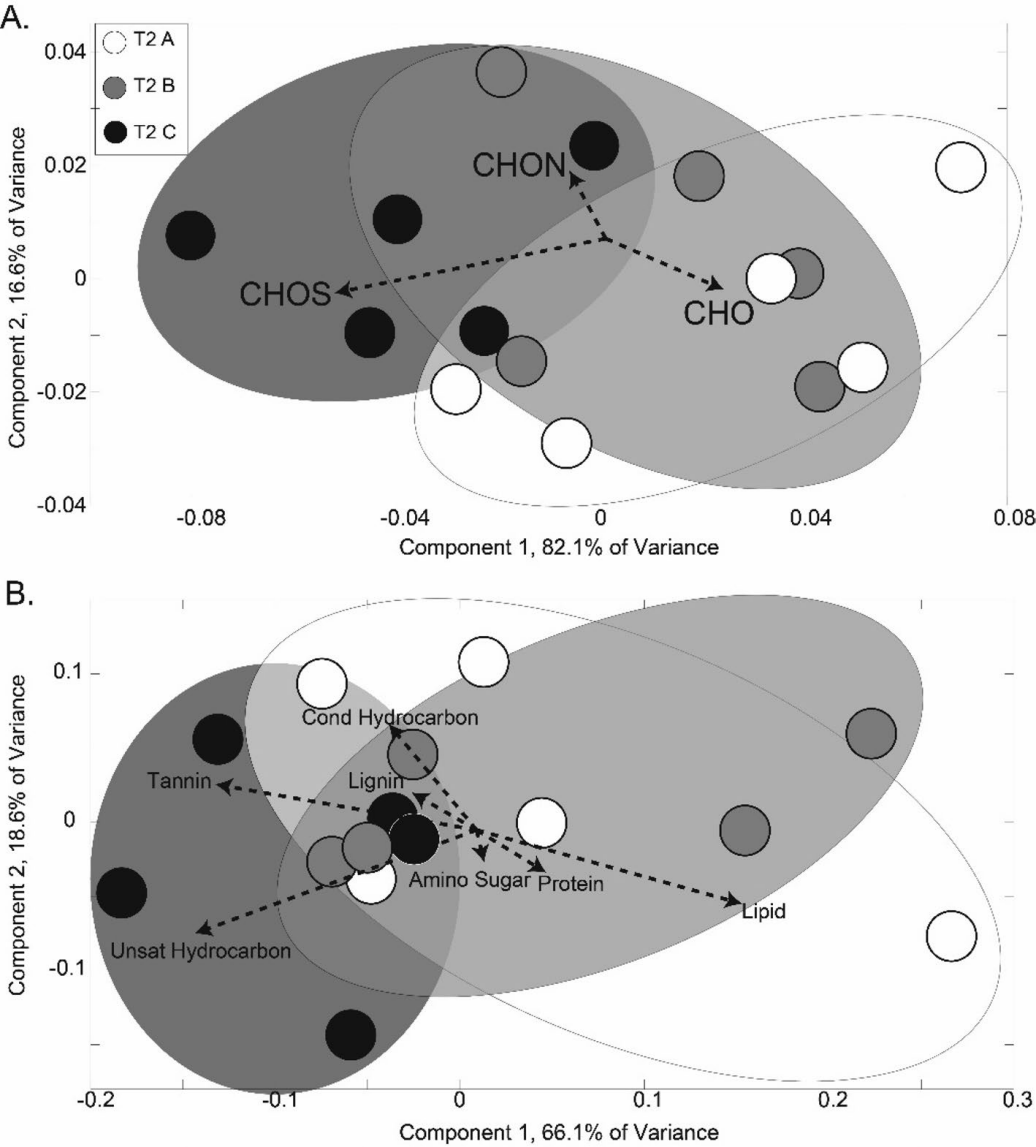
FTICR PCA plots exhibited the drought severity treatment gradient from treatment A (control), B (mild), to C (severe) at T2 by elemental composition (**A**) and compound class (**B**). Elemental composition class CHOS (**A**) and compound class tannins and unsaturated hydrocarbons (**B**) were associated with treatment C at T2 (after treatment). FTICR PCA plots comparing treatments A, B, and C at T1 (before treatment) are presented in Fig. S8. Arrows are vectors whose length indicates the loading score and how strongly each compound class influences a principal component. Angles between the vectors that are less than 90° represent positive correlation and above 90° represent negative correlation.

**Figure 5:**
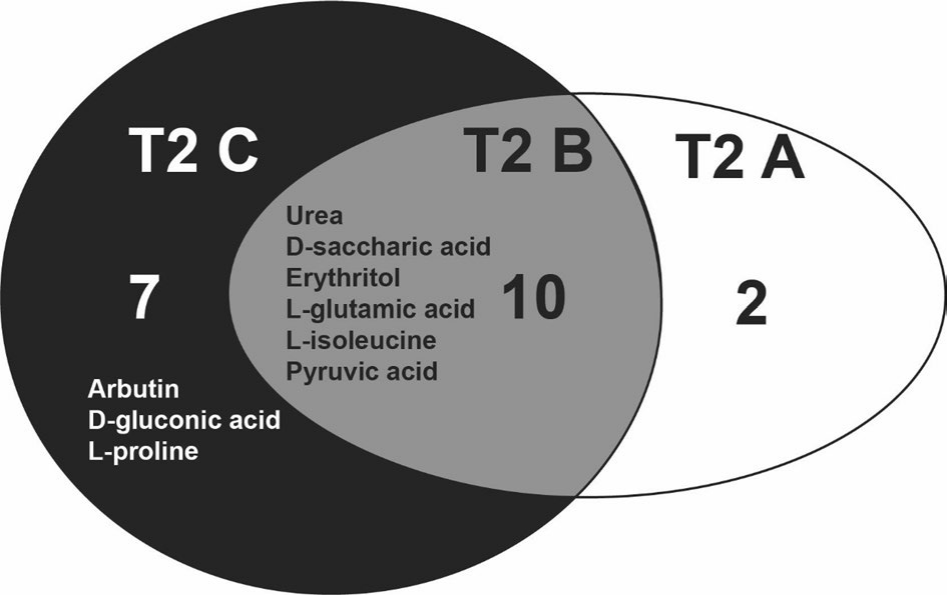
Unique root exudate compounds were detected within each treatment A (control), B (mild), and C (severe) at T2 (after treatment) using GC–MS. In total, 19 identified and non-identified metabolites were observed in treatments A, B, and C at T2 only (not at T1 (before treatment)). Identified metabolites unique to each treatment are listed while unidentified compounds are not listed (e.g. two compounds for A were not listed because they were not identified). Treatment B shared 10 compounds (6 identified and listed) with treatments A and C. Venn diagram of all (identified and unidentified) GC–MS metabolites found at T2 (including compounds from T1) are presented in Fig. S9.

**Figure 6:**
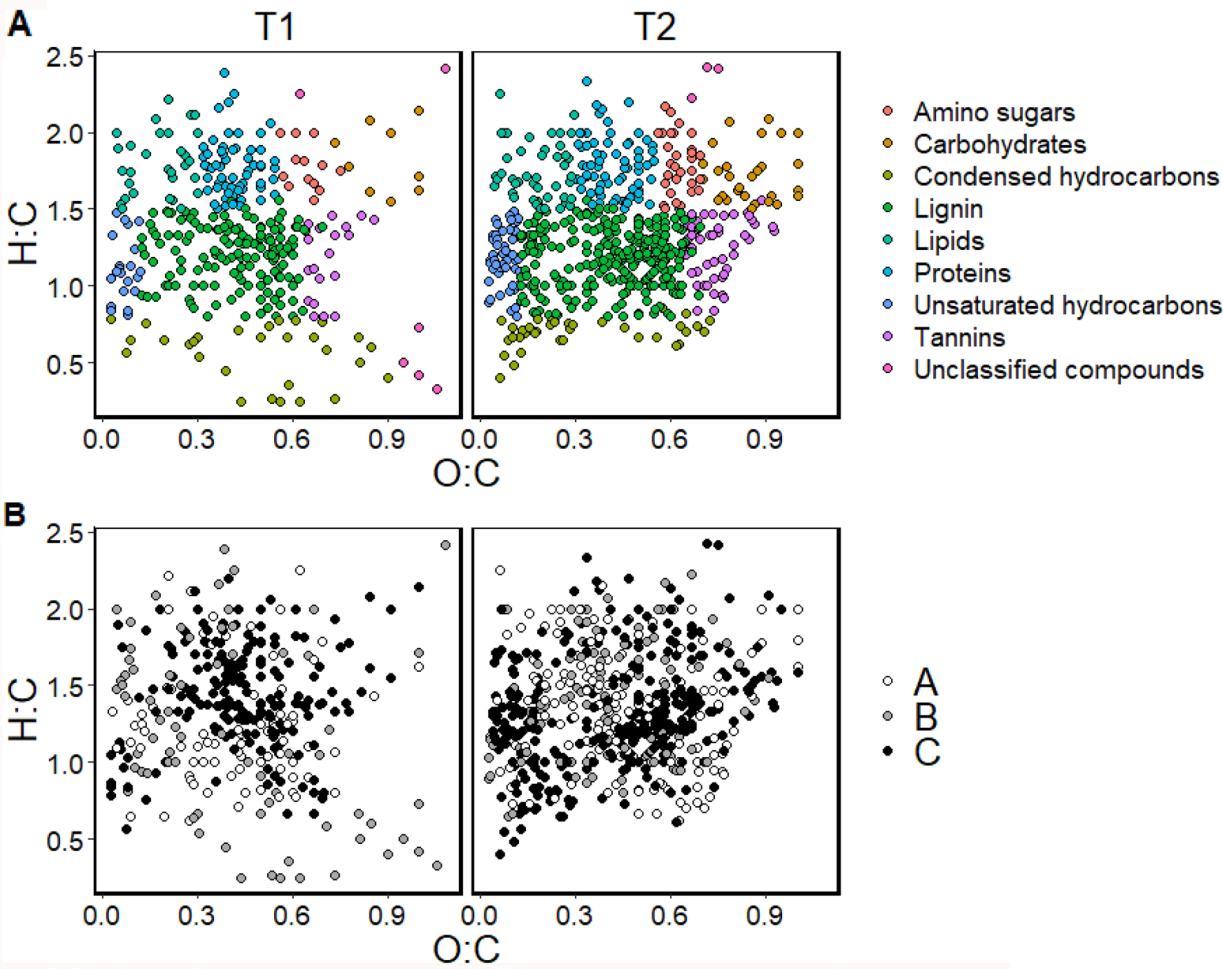
Root exudate composition increased from T1 (before treatment) to T2 (after treatment) (**A**), and shifted among treatments A (control), B (mild), and C (severe) between T1 and T2 (**B**). Van Krevelen plots of FTICR-detected compounds at T1 and T2 colored by compound class (**A**) and treatment (**B**). Van Krevelen plots depict the elemental ratios of O:C and H:C of compounds on the x and y axes, respectively, and are used to group compounds into high-level chemical classes^120,121^.

Similar to the physiological and root exudate C concentration measurements, root exudate composition reflected the gradient of drought severity treatments at T2, as indicated by consistent treatment patterns observed across the three independent analytical platforms used: GC–MS, NMR, and FTICR (Figs. 2, 3, 4, 5, 6). Similar to physiology and root exudate quantity (Fig. 1), the percent difference in normalized peak area between T1 and T2 for GC–MS- and NMR-identified compounds increased from treatment A to B to C (with increasing drought severity) (Fig. 2a,b). Additionally, mean normalized peak area for GC–MS- and NMR-identified metabolites significantly increased from treatment A to B to C (with increasing drought severity) at T2, but not at T1 (Supplementary Tables S3, S4). The same trend was observed in raw peak areas for GC–MS-identified metabolites of individual replicates (Supplementary Fig. S1), and mean normalized peak areas for NMR-identified metabolites (Supplementary Fig. S2) and for both identified and non-identified GC–MS metabolites (Supplementary Fig. S3). Overall, GC–MS detected 206 metabolites (75 were identified, 131 were unidentified), NMR detected 13 compounds, and FTICR identified 1300 to 2400 elemental formula assignments per sample (Supplementary Figs. S1, S2).

The percent change in GC–MS z-scores between T1 and T2 mirrored the drought severity gradient and increased from A to B to C in 64 of 206 GC–MS-detected metabolites (32 of the 64 were identified), and decreased from A to B to C in 28 of 206 GC–MS-detected metabolites (8 of the 28 were identified) decreased from A to B to C (Fig. 2c). The percent change in NMR z-scores between T1 and T2 also mirrored the drought severity gradient and increased from A to B to C in 5 of 13 NMR-detected metabolites, and decreased from A to B to C in 3 of 13 NMR-detected metabolites (Fig. 2d). The GC–MS compounds in Fig. 2c are listed in Supplementary Table S5.

A greater percentage of GC–MS- and NMR-identified metabolites at T2 had z-scores that mirrored the drought severity gradient and increased or decreased from treatment A to B to C than at T1 (Fig. 2, Supplementary Fig. S1, Supplementary Tables S5, S6, S7). Specifically, 44% of GC–MS-detected metabolites (i.e. 68 + 12 = 80 of 206) and 69% of NMR-identified metabolites (i.e. 5 + 4 = 9 of 13) increased or decreased at T2 from A to B to C, while at T1 only 26% of GC–MS-detected metabolites (i.e. 38 + 16 = 54 of 206) and 38% of NMR-identified metabolites (i.e. 5 + 0 = 5 of 13) showed this pattern (Supplementary Fig. S1, Supplementary Tables S6, S7). GC–MS-identified metabolites that increased or decreased at T1 and T2 are listed in Supplementary Table S6. NMR-identified metabolites that increased at T2 from A to B to C are listed in Supplementary Table S7 and include: sucrose, malic acid, glucose, betaine, and fumaric acid, while those that decreased at T2 from A to B to C were: valeric acid, 3-hydroxybutyric acid, butyric acid, and capric acid (Supplementary Table S7). Together, root exudate metabolite composition reflected a drought severity gradient from control (A) to mild (B) to severe (C), but the direction of the shifts depended on the specific metabolite.

This gradiential increase/decrease in specific compounds from A to B to C was also observed in GC–MS and NMR pPCAs, which showed that at T2, treatments A and B exhibited little separation, while both treatments A and B distinctly separate from treatment C (Fig. 3, Supplementary Figs. S4, S5, S6, S7). In contrast to T2, as expected, less separation among treatments was observed at T1 (Fig. 3, Supplementary Figs. S4, S5, S6). FTICR results revealed this same pattern of consistently greater separation at T2 between A and C and less separation between A and B, and between B and C (Fig. 4), that was not observed at T1 (Supplementary Fig. S8). At T2, treatment C separated from A and B based on unsaturated hydrocarbons and tannins, and on CHOS compounds (Fig. 4A,B). At T2, the normalized amount of unsaturated hydrocarbon compounds was the only compound class that significantly differed among treatments A, B, and C (*p* = 0.037), with treatment C significantly differing from treatment B (*p* = 0.041) and marginally differing from treatment A (*p* = 0.0987). At T2, the normalized amount of CHO, CHONS, and CHOS compounds significantly differed among treatments A, B, and C (*p* = 0.005, 0.047, 0.049, respectively), while CHON did not (*p* > 0.05). CHO of treatment C significantly differed from both treatments A (*p* = 0.005) and B (*p* = 0.034). CHONS of treatment C marginally differed from both treatments A (*p* = 0.090) and B (*p* = 0.062). CHOS of treatment C marginally differed from both treatments A (*p* = 0.062) and B (*p* = 0.098).

The top 10 GC–MS-identified metabolites driving T2 separation among treatments for the positive scores of principal component (PC) 1 were: sucrose, myo-inositol, mannose, arabinose, sedoheptulose anhydride monohydrate, pyruvic acid, tagatose, D-glucose, L-threonine, and 4-guanidinobutyric acid, while the top 10 identified metabolites for the negative scores were: adipic acid, 3-aminopropionitrile, 1-hexadecanol, L-serine, 1-methylhydantoin, benzoic acid, porphine, uracil, citraconic acid, and 3-hydroxy-3-methylglutaric acid (dicrotalic acid) (Fig. 3). In the NMR pPCA, the top identified metabolite driving T2 separation for the positive scores of PC 2 was butyric acid, while the top identified metabolites for the negative scores of PC 2 driving T2 separation were sucrose, betaine, and fumaric acid (Supplementary Fig. S7).

T2 contained 19 GC–MS-detected compounds not observed at T1, with 2 unidentified compounds unique to treatment A, 10 compounds present in all three treatments (6 identified), and 7 compounds unique to treatment C (3 identified) at T2 (Fig. 5). The 10 shared compounds contained 6 identified compounds: urea, D-saccharic acid, erythritol, L-glutamic acid, L-isoleucine, and pyruvic acid (Fig. 5). The 7 compounds unique to treatment C contained 3 identified compounds: arubtin, D-gluconic acid, and L-proline (Fig. 5; see also Supplementary Fig. S9 for a Venn diagram of all compounds (identified and unidentified) at T2 including compounds present at both T2 and T1). In contrast to GC–MS, NMR metabolites unique to each treatment were not observed. Van Krevelen diagrams of FTICR-detected compounds showed that the composition of high-level chemical classes shifted between T1 and T2 and among treatments A, B, and C (Fig. 6).

Many of the root exudate compounds and compound classes associated with increasing drought severity may alter plant drought responses and the rhizosphere microbiome (Table 2). These root exudate compounds most strongly associated with the separation of treatments at T2 as detected with GC–MS (Fig. 3, Supplementary Figs. S4, S5) and NMR (Supplementary Figs. S6, S7), and included CHOS, unsaturated hydrocarbons, and tannins (Fig. 4). Compounds unique to treatment C (Fig. 5) serve various functions in altering plant drought responses and the rhizosphere microbiome (Table 2, see Discussion).

**Table 2:** Name, type, and function of root exudate compounds linked to drought severity treatment and to the separation of treatments A (control), B (mild), and C (severe) at T2 (after treatment).

Name	Type	Function	References
Adipic acid, fumaric acid, butryic acid, 3-hydroxy-3-methylglutaric acid (dicrotalic acid, aconitic acid, caprylic acid, malonic acid	Carboxylic acid	Common in root exudates and serve as microbial nutrients	^20,122^
Aminopropionitrile	Organic compound	Rare compound observed in root exudates	^123^
Benzoic acid	Carboxylic acid	Observed in root exudates and has exhibied antifungal and allelopathic properties	^124,125^
Guanidinobutyric acid	Betaine	N compounds common in root exudates and ubiquitous in plants for their protective action in response to abiotic stress	^122,126^
Hexadecanol	Alcohol	Observed in root exudates of tall fescue and varied with tall fescue cultivar and endophyte presence	^127^
Myo-inositol	Sugar	Involved in signalling, sugar metabolism, and abiotic stress tolerance	^128,129^
Proline, serine, L-isoleucine, threonine, glutamic acid, valine	Amino acid	Serves as microbial chemoreceptors and may be a form of communication with microbes or a response to the presence of microbes, contributes to chemical defense against abiotic stressors (e.g. drought, cold, salt)	^20,130,131^
Sucrose, mannose, arabinose, glucose, galactose	Sugar	C sources for microbes, stimulate microbial activity, release in root exudates increased under drought	^60,61^
Tagatose	Sugar	Can inhibit the growth of some plant pathogens	^132,133^

Compounds were identified with GC–MS (Figs. 4, S4, S5) and NMR (Figs. S6, S7).

## Discussion

### Drought severity-induced shifts in physiology and root exudate C

In support of our first hypothesis, the increase in root exudate TC and TOC and concurrent decrease in predawn leaf water potential and photosynthesis at T2 with increasing drought severity suggested that drought severity increased plant C allocation to root exudate C. Significant declines in predawn leaf water potential and photosynthesis with increasing drought severity demonstrated the effectiveness of the drought severity treatments, and suggest that C allocation to root exudates was prioritized despite reduced C uptake.

Whether C is passively or actively allocated to different plant functions (e.g. root exudates, growth, storage) under stress is debated^55^. Our results suggest that individual plants under more severe drought may actively allocate C to root exudates even when C assimilation and growth presumably have stopped. While root exudation may not increase under drought in all species, our results are consistent with previous studies where the root exudation rate of monocots (e.g. blue grama) and other species increased during drought^11,28,29,56^.

The observed increase in C allocation to root exudates with increasing drought severity may be a mechanism to alter the rhizosphere microbiome because symbiotic microbes can benefit plant hosts under drought by enhancing water and nutrient acquisition^19,27,57^. Shifts in plant C allocation and root exudate concentration can alter soil microbial community composition^18,58,59^ and can stimulate the activity of soil microbes in the rhizosphere compared to bulk soil^20,60,61^.

Mean root:shoot did not significantly differ among treatments at T2, while total (root + shoot) biomass declined with increasing drought severity, suggesting that drought resulted in declines in both above- and belowground biomass of blue grama, similar to that observed by Zhen and Schellenberg^62^. This contrasts with observations that root:shoot ratio increases in response to drought reflecting greater C allocation to roots than shoots to access deeper water or nutrient sources^60,63^. However, increased root branching could lead to increases in root surface area without increases in total biomass^64^. Alternatively, drought severity may outweigh this effect if prolonged drought reduced root numbers, spread, and depth of penetration in blue grama root systems^65^. The lowest root:shoot in the severe drought treatment (albeit root:shoot did not significantly differ among T2 treatments) may indicate root mortality due to the severe drought^30^ (only live roots attached to the plant were measured). Additionally, blue grama belowground allocation under drought may be more nuanced, which would be similar to other grass species in which drought affected crowns and rhizomes moreso than roots and shoots^33^. In this study however, we did not separate crowns and rhizomes nor measure root branching.

### Drought severity-induced shifts in root exudate composition

Further supporting our first hypothesis, we observed a gradient of shifts in root exudate composition as measured with three independent analytical platforms (GC–MS, NMR, FTICR) that mirrored the gradient of drought severity treatments (control to mild to severe) measured by predawn leaf water potential and photosynthesis. Percent changes in GC–MS and NMR peak areas and z-scores exhibited an increasing or decreasing trend from treatment A to B to C at T2—trends not observed at T1 (Fig. 2, Supplementary Tables S6, S7). We also observed GC–MS pPCA separation and FTICR PCA separation reflecting the drought severity treatment gradient at T2 and not at T1 (Figs. 3, 4, Supplementary Fig. S8). Together, this suggests that root exudate composition can be modulated by drought stress severity^56,66^. For example, the intermediate severity of treatment B induced intermediate drought-related changes in root exudate composition compared to treatments A (control) and C (severe). Supporting our findings, root exudate composition has also been observed to differ between plants under drought, plants recovering from drought, and non-droughted plants^31^. However, to our knowledge, no study has investigated the effect of drought severity on blue grama root exudate composition.

In our study, root exudate composition reflected increasing drought severity possibly because specific root exudate compounds can promote drought resistance and/or support a rhizosphere microbiome that may promote drought resistance. In support of our second hypothesis and described below, we found that the specific compounds and compound types that were driving the T2 treatment separation and/or were unique to the severe drought treatment, also have been observed to alter plant drought responses and the rhizosphere microbiome (Table 2). Identifying specific compounds linked to increasing drought severity with multiple analytical platforms (GC–MS, NMR, FTICR) helped us to elucidate mechanisms of root exudation that may be compound-specific^20^.

### Identification of compounds that affect plant drought responses

Plants produce compounds that promote drought resistance mechanisms including: providing hydrophobic protection against drying soil^30,67^, accumulating osmotically active solutes to maintain turgor (e.g., proline), providing antioxidant defense^68,69^, enhancing root growth, and stress hormone signaling^24,25^. Consistent with this, we observed an increase in drought-resistance-promoting compounds in root exudates with increasing drought severity (Table 2). For example, following drought treatment (T2), proline and D-gluconic acid were two of three identified GC–MS metabolites unique to treatment C (Fig. 5, Supplementary Figs. S4, S5), suggesting an increase in production of compounds associated with drought protection with increasing drought severity. These organic acids in root exudates can solubilize inorganic phosphates and enhance N availability, directly promoting plant growth^15,70–72^. Proline, an amino acid, is well-known as an osmolyte and osmoprotectant in plant drought response^30^, and can also contribute to cellular homeostasis, redox and antioxidant functions, and signalling^73–77^. Consistently, proline has been observed to increase in root exudates under drought^30^, and Bokhari et al.^47^ observed that proline composed the majority of amino acids in the root exudates of greenhouse-grown blue grama. Our observed drought severity-induced association with proline, a N-rich compound, is consistent with the drought-severity-induced association with CHOS, CHON, and CHONS compounds, driving the separation between drought severity treatments (Fig. 4). CHONS compounds also may be linked to the release of mucilage (proteins, extracellular DNA) used for defense against pathogens, lubricating the root zone, and stabilizing soil particles^20,78^. An increase in N-rich compounds in root exudates with increasing drought severity may indicate a surplus N being released by roots^38^ or reallocation of N belowground. N compounds in root exudates may be related to the observed decline in photosynthesis with drought severity because N is the most prevalent element in RUBISCO, the enzyme responsible for photosynthesis (Chapin ^134^). In addition to CHONS, CHOS compounds influenced treatment separation at T2, suggesting that the release of sulfur-containing compounds in root exudates also may be related to plant response to drought severity. Sulfur-containing compounds are often secondary metabolites that contribute to plant defense and stress response, yet not much is known about them^79^.

### Identification of compounds that affect the rhizosphere microbiome

Root exudate compounds most strongly associated with treatment separation at T2 as detected with GC–MS (Fig. 3, Supplementary Figs. S4, S5) and NMR (Supplementary Figs. S6, S7) have been commonly observed in root exudates to alter the rhizosphere microbiome (Table 2)^17,18,27,80^. In contrast to compounds that attract microbes, some root exudate compound types such as arbutin or compounds with a catechol ring^61^ may be toxic and/or reduce microbial activity, consequently repelling certain microbes. Arbutin was one of the three identified GC–MS metabolites unique to T2 treatment C (Figs. 2, 5, Supplementary Figs. S4, S5), suggesting an increase in production of arbutin associated with increasing drought protection with increasing drought severity.

The microbial modulation roles of unsaturated hydrocarbons and tannin-like compounds, observed to drive the separation between drought severity treatments at T2 (Fig. 4), are not well understood partly because these compounds have great structural diversity, and consequently have diverse effects on soil microbes^61,81,82^. Tannins are polyphenols, which may bind N and proteins to form recalcitrant complexes, affecting the soil microbial community and nutrient cycling^81^. Polyphenolic compounds such as tannins can influence surrounding plant and microbial growth and development by functioning as a toxin^83^, food source, microbial attractant^84^, or allelopathic, chemotactic, and signaling molecules^85–88^; and influencing enzymatic activity^89–91^ and drought and pathogen stress response^92^. Like tannins, unsaturated hydrocarbons and polyphenolics are also often secondary metabolites which are associated with plant defense response^84,93^, and may increase in root exudates in response to drought^31^ and under stress^94,95^. Unsaturated hydrocarbon secondary metabolites include monoterpenes and other volatile terpenes^96^.

Future work is needed to understand the interlinked effects of drought on plant C allocation, root exudation, and interactions with the rhizosphere. However, several challenges exist and require further investigation. First, root exudate collection methods, especially in natural soil environments (versus hydroponics) are notoriously challenging^20,97,98^. Second, in addition to environmental stress, root exudation varies with plant age, development stage^99^, genotype^100^, species^61,101–103^, residence time of plants in soil, stress type^28^, and root tissue type (e.g. primary and lateral root, root tip)^20,104^. Third, root exudates contain a vast diversity of compounds requiring the use of multiple metabolomic platforms^56^. Research that considers how these factors influence drought-induced impacts on plant C allocation, root exudates, and the plant microbiome will improve predictions of terrestrial C fluxes under future climate regimes and tools to engineer beneficial plant–microbe interactions to enhance plant performance during stress.

## Materials and methods

### Experimental setup

Blue grama was grown from seed (Wind River Seed Co., Manderson, WY, USA) in a greenhouse (Bozeman, MT, USA) in small trays in a 50:50 mix of sand:soil collected from the top soil layer (0–10 cm) of a field site (Bozeman, MT, USA) where blue grama naturally grows (e.g. Badri et al.^87^). This ensured microbes relevant to blue grama would be available^27^. Permission was obtained to collect soil and all study protocols involving plant material were conducted in accordance with institutional, national, and international guidelines and legislation. Seeds were planted October 1, 2019. After 30 days of initial growth under well-watered conditions, 120 seedlings of similar size (including their growing soil) were transplanted to larger pots (0.75 L) of sterilized sand (e.g. Ulrich et al.^12^). After transplanting, plants were watered to field capacity and allowed to acclimate to greenhouse conditions for an additional 30 days. Then, established plants were divided into three treatment groups: control (treatment A), mild drought (treatment B), and severe drought (treatment C) (n = 40 plants per treatment) (Supplementary Fig. S10). The control treatment group received watering every other day to maintain field capacity. The mild drought treatment group was watered half as frequently to impose a mild drought. The severe drought treatment group was watered 25% as frequently to impose a severe drought. Treatments lasted 30 days. These drought severity levels were selected based on our previous work^12^. Here, we selected less severe drought treatment levels to induce plant stress response without killing the plants, allowing us to capture shifts in physiology and root exudate concentration and composition as a function of drought severity. Greenhouse conditions during the study (Oct–Jan 2019) consisted of a 16-h photoperiod, daytime temperature of 23.8 °C, nighttime temperature of 21.1 °C, and average daytime photosynthetically active radiation of 500 μmol m^2^ s^−1^.

### Measurements and root exudate collection

To determine how drought severity influenced plant physiology, we measured predawn leaf water potential, leaf gas exchange (photosynthesis, stomatal conductance), root exudate concentration and composition, and root and shoot biomass to determine root to shoot biomass ratios (root:shoot) and total biomass in each treatment group before (T1) and after (T2) 30 days of treatment (Dec 19–Jan 18). All measurements were made on the same 5 randomly selected individuals per treatment and within one week before the treatment start date (T1) and within one week after the treatment end date (T2). Predawn leaf water potential was measured using a Scholander type pressure chamber (1505D, PMS Instruments, Corvallis, OR, USA). Gas exchange was measured using a portable photosynthesis instrument equipped with an infrared gas analyzer (LI-6800, Licor, Lincoln, NE USA) on five plants randomly selected from each treatment group. Cuvette conditions were set to: 1000 µmol m^−2^ photosynthetic photon flux density, 60% relative humidity, 400 ppm [CO_2_], 30 °C leaf temperature, and 500 μmol s^−1^ flow rate.

To determine how drought severity treatment influenced root exudate concentration and composition and allow for pairing with plant physiological measurements, we collected root exudates before (T1) and after (T2) treatment from the same 5 plants per treatment used for gas exchange and leaf water potential measurements using the^105^ method (e.g.^28,29^). Briefly, plants were excavated and roots were rinsed of soil in DI water and dipped in an antimicrobial solution to halt the microbial production of C compounds. This ensured that we collected exudates from the plant and not microbes. Plants were subsequently transplanted to filter flasks of glass beads that do not provide a C source but still provide mechanical pressure on the root system, resembling natural soil conditions. Plant roots were flushed with 100 mL of sterile water using a vacuum pump connected to the filter flasks, another 100 mL was added, and plants were allowed to release exudates over a 41-h period. The remaining solution was filtered (0.22 µm) and collected for root exudate concentration and composition analyses. Root exudates were frozen and stored at − 80 °C until analysis. After exudate collection, plants were destructively harvested for root and shoot biomass measurements. We recognize that the^105^ method to extract exudates disturbs plants. However, we had successfully tested the method with loblolly pine seedlings^106^. The method has been successful in similar experiments^28,107^, and benefitted our study because it allowed: the collection of exudates without interference from microbial consumption; sampling all exudate types from the entire root system rather than only specific compounds from a small area of roots; and collection from soil as opposed to hydroponic systems that can underestimate root exudation^108^.

### Root exudate analysis

Root exudate concentration and composition were analyzed at the Environmental Molecular Sciences Laboratory of the US Department of Energy’s Pacific Northwest National Laboratory. Samples were thawed, prepared, and analyzed as described below. Total organic carbon (TOC) (root exudate C concentration) was measured (Elementar VarioTOC Cube TOC Analyzer, Elementar Analysensysteme Langenselbold, Germany). TOC was determined by the difference between the measured total carbon (TC) and the measured total inorganic carbon (TIC). Root exudate composition was characterized using gas chromatography-mass spectrometry (GC–MS), 12-Tesla Fourier Transform Ion Cyclotron Resonance (FTICR) mass spectrometry, and nuclear magnetic resonance (NMR). Using multiple instruments provides a more comprehensive characterization of the root exudates than one instrument alone because each instrument has its disadvantages and advantages^109^. For example, NMR can only characterize a relatively small number of identified compounds. GC–MS can detect both identified and unidentified compounds. FTICR is highly sensitive and accurate and can detect the greatest number of unique compounds, and although it cannot identify all detectable compounds, FTICR can classify compounds by their elemental composition (C, H, O, N, S, P) or compound class type (e.g. lipids, sugars, protein).

### GC–MS

Dried metabolite extracts from samples were derivatized using a modified version of the protocol used to create FeihnLib^110^. Samples underwent methoximation to protect carbonyl groups and reduce tautomeric isomers, followed by silylation with N-methyl-N-trimethylsilyltrifluoroacetamide and 1% trimethylchlorosilane (MSTFA) to derivatize hydroxy and amine groups to trimethylsilated (TMS) forms. Samples were then analyzed by an Agilent GC 7890A coupled with a single quadrupole MSD 5975C (Agilent Technologies, Santa Clara, CA, USA) over a mass range of 50–550 m/z to identify molecular masses present. To identify metabolites, GC–MS raw data files were processed using Metabolite Detector software, version 2.5 beta^111^. Retention indices of detected metabolites were calculated based on analysis of the fatty acid methyl esters (FAMES) standard mixture followed by chromatographic deconvolution and alignment. Metabolites were initially identified by matching experimental spectra to an augmented version of FiehnLib^112^. All metabolite identifications were manually validated with the NIST 14 GC–MS library. Summed abundances of the three most abundant fragment ions of each identified metabolite were integrated across the GC elution profile (determined by Metabolite Detector). Fragment ions due to trimethylsilylation were excluded from the determination of metabolite abundance. Features resulting from GC column bleeding were removed from the data before further analysis. One sample at T1 treatment A had very low signal and was removed as an outlier. Metabolites that were present in more than one replicate were included in Venn diagram comparing metabolites across treatments.

### NMR

Lyophilized material was resuspended in 300 µL of 0.5 mM sodium 2,2-dimethyl-2-silapentane-5-sulfonate-d6 (DSS-d6) in 99.98% D_2_O and thoroughly mixed prior to transfer to a 3-mm Standard Bruker NMR tube. NMR spectra were acquired on a Varian 600 MHz VNMRS spectrometer equipped with a 5 mm triple-resonance (HCN) cryogenically cooled probe with the sample analyzed at a regulated temperature of 298 K. One-dimensional 1H spectra were acquired using a Nuclear Overhauser Effect spectroscopy (NOESY) pulse sequence with a spectral width of 12 ppm and 512 transients. The NOESY mixing time was 100 ms and the acquisition time was 4 s followed by a relaxation delay of 1.5 s during which presaturation of the water signal. NMR raw data files were processed using NMRPipe^113^. Time domain free induction decays (57,792 total points) were zero filled to 131,072 total points prior to Fourier transform. Chemical shifts were referenced and normalized to the DSS-d6 at 0 ppm. The 1D ^1^H spectra were manually processed and peak picked. Assignments for metabolite identifications were made using Chenomyx NMR Suite 8.5 and BioMagResBank^114^. Metabolite identification was based on matching the chemical shift, J-coupling, and intensity of experimental signals to compound signals in the Chenomyx database when applicable.

### FTICR

Samples were extracted by the solid phase extraction method used in Dittmar et al.^115^ and Tfaily et al.^116^ where samples were acidified to a pH of 2, run through a Bond Elut PPL cartridge, and were eluted off with MeOH (total volume recovered was 1 mL). Samples were then stored at − 80 °C. Samples were infused into the FTICR mass spectrometer by an automated direct injection system at a flow rate of 3 µl/min^116^. Samples were co-added for 144 scans, 100–900 Da. Spectra were inspected and reruns were appended to the queue. Samples were peak picked using Bruker DA software with a signal to noise ratio of 7, the standard cutoff for Bruker FTICR data. Data were calibrated and formula assigned using in-house software Formularity^117^. About 2000 peaks or compounds were assigned per sample. FTICR classified all compounds by elemental composition and compound class. Elemental class or composition (molecular formula) detected included: CHO, CHON, CHOS, CHONS, and CHOP. CHOP assignments, however, were ambiguous because of Cl in samples even with PPL (Priority PolLutant) clean up. Compound classes detected were amino sugar, carbohydrate, condensed hydrocarbon, lignin, lipid, protein, tannin, and unsaturated hydrocarbon.

### Statistical analyses

Linear models (LM) were fit to determine the effects of drought severity on mean physiological response variables: predawn leaf water potential, photosynthesis, stomatal conductance, root to shoot biomass ratio (root:shoot), total (root+shoot) biomass, root exudate total inorganic carbon (TIC), root exudate total organic carbon (TOC), and root exudate total carbon (TC; TC = TIC + TOC). Diagnostic plots were used to confirm that the LM assumptions of equal variance, normality of residuals, and influential points were met. TC, TOC, TIC, and root:shoot data were log transformed to meet assumptions of normality based on quantile–quantile plots. One-way analysis of variance (ANOVA) was conducted across all drought treatments at T1 for each mean physiological response variable to confirm lack of mean treatment group differences at T1 (before treatment). Means that did not significantly differ among T1 drought treatments were averaged together to provide a baseline to determine the percent difference (i.e. percent change) between T1 and after treatment (T2) for each replicate of all response variables. Mean percent difference or change between T1 and T2 for each individual was determined. One-way ANOVA was used to identify signficant differences in means among treatments A, B, and C at T2 for each response variable. General Linear Hypothesis Tests adjusted for type one error inflation were then fit on significantly different one-way ANOVAs as a part of the Tukey multiple comparison procedure using the library *multcomp*^118^. Tukey’s Honest Significant Difference (HSD) test was then used to identify significant pairwise treatment differences using a 95% confidence interval.

Processed GC–MS peak areas were analyzed in R version 3.6.3. Processed NMR peak areas were peak picked and analyzed in MATLAB 2019b. For both GC–MS and NMR, all 0 s were replaced with NaN and log_10_ transformed with a global normalization to the median. For both GC–MS and NMR, probabilistic Principal Components Analysis (pPCA) and ANOVA were performed to identify significant treatment differences and differences within treatments between T1 and T2. pPCA identified the top 10 compounds that explained the variability in the data associated with the positive and negative. For both GC–MS and NMR, z-scores on raw peak areas were calculated as z-score = $\frac{{{\text{x}} - {\text{X}}}}{{\text{S}}}$, where x is a peak area value of an individual sample, X is the mean peak area across the samples, and S is the standard deviation of the individual value across the samples. Z-score helps to visualize the differences between values because z-score is relative to the range of values of the replicates. pPCA takes into account missing values, which we had in the GC–MS and NMR data. We did not have that issue with the FTICR data.

Processed FTICR data were analyzed with Principle Components Analysis (PCA) plots and heat maps generated using MetaboAnalyst 5.0 (https://www.metaboanalyst.ca/)^119^ using counts of elemental composition (molecular formula) and compound class as defined by a Van Krevelen diagram. A Van Krevelen diagram, one of the most common ways to visualize FTICR datasets, plots the elemental ratios of O:C and H:C of compounds on the x and y axes, respectively, and is used to group compounds into high-level chemical classes^120,121^. The number of peaks of each elemental class and compound class were normalized to the total number of peaks in the sample (i.e. total number of formulas assigned) to determine percent of peaks of each elemental class and compound class (i.e. normalized quantities of each elemental class and compound class). One-way ANOVA was used to identify signficant differences in percent of peaks of each elemental class and compound class among treatments at T2. Tukey’s HSD test was used to identify significant pairwise comparisons.

## Supplementary Information

Supplementary Information.

## Data Availability

Root exudate metabolomics data are publicly and freely available through Mass Spectrometry Interactive Virtual Environment (MassIVE): Accession: MSV000088504, URL: https://massive.ucsd.edu/ProteoSAFe/dataset.jsp?task=993f4d04def14767af9c0452ad6808ae. Other data that support the findings of this study are available from the corresponding author upon reasonable request.
