# Supplementary material for: Root exudate composition reflects drought severity gradient in blue grama (Bouteloua gracilis)
Source: Sci Rep. 2022 Jul 22;12:12581. doi: 10.1038/s41598-022-16408-8 (PMC9307599; doi:10.1038/s41598-022-16408-8)
Supplement: Supplementary file 1 — Supplementary Information. [file 41598_2022_16408_MOESM1_ESM.docx]

**Supplementary Information**

**Table S1** Results from Tukey’s HSD identifying significant differences in treatment means at T2 for each physiological response variable (Fig. 1).

**Table S2** Mean, sample size (N), standard deviation (SD), and standard error (SE) of physiological measurements.

**Table S3** ANOVA table identifying significant differences between treatment means at T1 and T2 for GC-MS and NMR mean normalized peak area (Figs 2**a**, **b**).

**Table S4** Results from Tukey’s HSD identifying significant differences in treatment means at T1 and T2 for GC-MS and NMR mean normalized peak area (Figs 2**a**, **b**).

**Table S5** GC-MS compounds listed in the Fig. 2**c** and Supplementary Fig. S1**b** heat maps.

**Table S6** GC-MS-identified compounds whose z-scores reflected the drought severity treatment gradient and increased or decreased from control to mild to severe drought severity (treatments A to B to C) before (T1) and after treatment (T2).

**Table S7** NMR-identified compounds whose z-scores reflected the drought severity treatment gradient and increased or decreased from A to B to C before (T1) and after treatment (T2).

**Figure S1** GC-MS median of the raw peak areas for identified metabolites of individual samples (**a**) and GC-MS heat map of z-scores for individual samples and identified metabolites (**b**).

**Figure S2** NMR spectra, identified metabolites, chemical shifts, and mean normalized peak area for identified metabolites in each timepoint and treatment.

**Figure S3** GC-MS median of the raw peak areas for all metabolites (**a**) and only identified metabolites (**b**), and only identified metabolites that significantly differed between treatments (**c**).

**Figure S4** GCMS probabilistic principal component analysis of identified metabolites only (A) of all treatments at T1 and T2.

**Figure S5** GC-MS probabilistic principal component analysis on all identified and unidentified features of all treatments at T1 and T2.

**Figure S6** NMR probabilistic principal component analysis on identified metabolites of all treatments at T1 and T2

**Figure S7** NMR probabilistic principal component analysis of all treatments at only T2.

**Figure S8** FTICR principal components analysis plots comparing treatments A, B, and C at T1 by elemental composition (**a**) and compound class (**b**)

**Figure S9** Venn diagram of all (identified and unidentified) GC-MS metabolites found at T2

**Figure S10** Experimental design

**Table S1** Results from Tukey’s HSD identifying significant differences between treatment means at T2 for each physiological response variable (predawn leaf water potential, photosynthesis, stomatal conductance, root to shoot biomass ratio (root:shoot), root exudate total C (TC), root exudate total inorganic C (TIC), root exudate total organic C (TOC), total (root+shoot) biomass) including treatment comparison, the difference between treatment means, the lower and upper 95% family-wise confidence level, and p-value.

|  | **Treatment comparison** | **Difference between means** | **Lower 95% CI** | **Upper 95% CI** | **p-value** |
| --- | --- | --- | --- | --- | --- |
| **Predawn leaf water potential** | B-A | -30.44 | -77.90 | 17.02 | 0.24 |
|  | C-A | -70.58 | -118.04 | -23.12 | **<0.01** |
|  | C-B | -40.14 | -87.60 | 7.32 | 0.10 |
| **Photosynthesis** | B-A | -1.68 | -2.86 | -0.51 | **<0.01** |
|  | C-A | -2.21 | -3.32 | -1.10 | **<0.001** |
|  | C-B | -0.53 | -1.70 | 0.64 | 0.47 |
| **Stomatal conductance** | B-A | 0.00 | -0.03 | 0.02 | 0.86 |
|  | C-A | -0.01 | -0.03 | 0.01 | 0.26 |
|  | C-B | -0.01 | -0.03 | 0.01 | 0.56 |
| **Root:shoot** | B-A | -0.61 | -1.51 | 0.29 | 0.21 |
|  | C-A | -0.24 | -1.14 | 0.66 | 0.76 |
|  | C-B | 0.37 | -0.53 | 1.27 | 0.54 |
| **Total (root+shoot) biomass** | B-A | -59 | -202 | 84 | 0.53 |
|  | C-A | -155 | -297 | -12 | **0.03** |
|  | C-B | -96 | -239 | 47 | 0.21 |
| **TC** | B-A | 0.26 | -0.35 | 0.87 | 0.50 |
|  | C-A | 0.99 | 0.38 | 1.60 | **<0.01** |
|  | C-B | 0.73 | 0.12 | 1.34 | **0.02** |
| **TIC** | B-A | 0.16 | -0.55 | 0.88 | 0.81 |
|  | C-A | 0.61 | -0.10 | 1.33 | 0.09 |
|  | C-B | 0.45 | -0.26 | 1.16 | 0.25 |
| **TOC** | B-A | 0.39 | -0.30 | 1.08 | 0.31 |
|  | C-A | 1.35 | 0.66 | 2.04 | **<0.001** |
|  | C-B | 0.96 | 0.27 | 1.65 | **<0.01** |

^Bold values indicate p<0.05. Letters in Figure 1 correspond with the results of this table.^

**Table S2** Mean, sample size (N), standard deviation (SD), and standard error (SE) of physiological measurements.

|  | **Timepoint** | **Treatment** | **Mean** | **N** | **SD** | **SE** |
| --- | --- | --- | --- | --- | --- | --- |
| **Predawn leaf water potential (MPa)** | T1 | A | -0.5720 | 5 | 0.1889 | 0.0845 |
|  | T1 | B | -0.5100 | 5 | 0.1962 | 0.0877 |
|  | T1 | C | -0.4740 | 5 | 0.1729 | 0.0773 |
|  | T2 | A | -0.3420 | 5 | 0.1361 | 0.0609 |
|  | T2 | B | -3.3860 | 5 | 3.9265 | 1.7560 |
|  | T2 | C | -7.4000 | 5 | 2.8810 | 1.2884 |
| **Photosynthesis (µmol m^-2^ s^-1^)** | T1 | A | 3.5359 | 5 | 2.2409 | 1.0022 |
|  | T1 | B | 3.3373 | 5 | 3.1964 | 1.4295 |
|  | T1 | C | 3.2716 | 5 | 1.7827 | 0.7972 |
|  | T2 | A | 1.8609 | 5 | 0.5680 | 0.2540 |
|  | T2 | B | 0.1805 | 4 | 1.0288 | 0.5144 |
|  | T2 | C | -0.3493 | 5 | 0.1997 | 0.0893 |
| **Stomatal conductance (mol m^-2^ s^-1^)** | T1 | A | 0.0212 | 5 | 0.0083 | 0.0037 |
|  | T1 | B | 0.0095 | 5 | 0.0053 | 0.0024 |
|  | T1 | C | 0.0106 | 5 | 0.0202 | 0.0090 |
|  | T2 | A | 0 | 5 | 0.0138 | 0.0062 |
|  | T2 | B | 0 | 4 | 0.0086 | 0.0043 |
|  | T2 | C | 0 | 5 | 0.0114 | 0.0051 |
| **Root:Shoot** | T1 | A | 2.0674 | 5 | 1.7483 | 0.7819 |
|  | T1 | B | 0.8742 | 5 | 0.1950 | 0.0872 |
|  | T1 | C | 1.3143 | 5 | 0.5331 | 0.2384 |
|  | T2 | A | 1.0255 | 5 | 0.2746 | 0.1228 |
|  | T2 | B | 0.7627 | 5 | 0.2259 | 0.1010 |
|  | T2 | C | 0.7231 | 5 | 0.4642 | 0.2076 |
| **Total Biomass (mg)** | T1 | A | 320.7000 | 5 | 238.9318 | 106.8535 |
|  | T1 | B | 158.1600 | 5 | 39.7555 | 17.7792 |
|  | T1 | C | 217.6000 | 5 | 82.1421 | 36.7351 |
|  | T2 | A | 314.7600 | 5 | 97.2996 | 43.5137 |
|  | T2 | B | 255.7600 | 5 | 89.8139 | 40.1660 |
|  | T2 | C | 159.9200 | 5 | 62.8144 | 28.0915 |
| **TC (mg/L/mg root biomass))** | T1 | A | 0.2023 | 5 | 0.1163 | 0.0520 |
|  | T1 | B | 0.3745 | 5 | 0.0949 | 0.0424 |
|  | T1 | C | 0.3988 | 5 | 0.1745 | 0.0781 |
|  | T2 | A | 0.1959 | 5 | 0.0812 | 0.0363 |
|  | T2 | B | 0.2559 | 5 | 0.1186 | 0.0530 |
|  | T2 | C | 0.5139 | 5 | 0.1500 | 0.0671 |
| **TIC (mg/L/mg root biomass)** | T1 | A | 0.1398 | 5 | 0.0853 | 0.0382 |
|  | T1 | B | 0.2513 | 5 | 0.0735 | 0.0329 |
|  | T1 | C | 0.2918 | 5 | 0.1452 | 0.0650 |
|  | T2 | A | 0.1234 | 5 | 0.0598 | 0.0268 |
|  | T2 | B | 0.1466 | 5 | 0.0763 | 0.0341 |
|  | T2 | C | 0.2234 | 5 | 0.0913 | 0.0408 |
| **TOC (mg/L/mg root biomass))** | T1 | A | 0.0626 | 5 | 0.0317 | 0.0142 |
|  | T1 | B | 0.1232 | 5 | 0.0272 | 0.0122 |
|  | T1 | C | 0.1070 | 5 | 0.0441 | 0.0197 |
|  | T2 | A | 0.0725 | 5 | 0.0241 | 0.0108 |
|  | T2 | B | 0.1092 | 5 | 0.0469 | 0.0210 |
|  | T2 | C | 0.2905 | 5 | 0.1148 | 0.0513 |
| **Root biomass (mg)** | T1 | A | 221.0600 | 5 | 225.8857 | 101.0192 |
|  | T1 | B | 73.4800 | 5 | 21.1218 | 9.4460 |
|  | T1 | C | 125.7200 | 5 | 69.1074 | 30.9058 |
|  | T2 | A | 157.8600 | 5 | 57.5882 | 25.7542 |
|  | T2 | B | 107.7400 | 5 | 37.1955 | 16.6343 |
|  | T2 | C | 56.4800 | 5 | 12.7897 | 5.7197 |
| **Shoot biomass (mg)** | T1 | A | 99.6400 | 5 | 20.5142 | 9.1742 |
|  | T1 | B | 84.6800 | 5 | 21.4692 | 9.6013 |
|  | T1 | C | 91.8800 | 5 | 14.6614 | 6.5568 |
|  | T2 | A | 156.9000 | 5 | 45.4844 | 20.3412 |
|  | T2 | B | 148.0200 | 5 | 61.1394 | 27.3424 |
|  | T2 | C | 103.4400 | 5 | 53.8913 | 24.1009 |

**Table S3** ANOVA table identifying significant differences between treatment means at T1 and T2 for GC-MS and NMR mean normalized peak area (Figs 2**a**, **b**).

|  | **T1** | | | | | **T2** | | | | |
| --- | --- | --- | --- | --- | --- | --- | --- | --- | --- | --- |
|  | **DF** | **SS** | **MS** | **F** | **p-value** | **DF** | **SS** | **MS** | **F** | **p-value** |
| **GC-MS peak area** |  |  |  |  |  |  |  |  |  |  |
| treatment | 2 | 0.000018 | 8.7e-06 | 1.03 | 0.39 | 2 | 0.14 | 0.067 | 43.25 | **<0.0001** |
| residuals | 12 | 0.00010 | 8.5e-06 |  |  | 12 | 0.02 | 0.0016 |  |  |
| **NMR peak area** |  |  |  |  |  |  |  |  |  |  |
| treatment | 2 | 0.011 | 0.0055 | 0.41 | 0.67 | 2 | 0.017 | 0.0085 | 24.74 | **<0.0001** |
| residuals | 12 | 0.16 | 0.013 |  |  | 11 | 0.004 | 0.00034 |  |  |

^Degrees of freedom (DF), sum of squares (SS), mean square (MS), F-statistic (F), p-value. Bold values indicate p<0.05.^

**Table S4** Results from Tukey’s HSD identifying significant differences between treatment means at T1 and T2 for GC-MS and NMR mean normalized peak area (Figs 2**a**, **b**) including treatment comparison, the difference between means, the lower and upper 95% confidence level, and p-value.

|  |  | **T1** | | | | **T2** | | | |
| --- | --- | --- | --- | --- | --- | --- | --- | --- | --- |
|  | **Treatment comparison** | **Difference between means** | **Lower 95% CI** | **Upper 95% CI** | **p-value** | **Difference between means** | **Lower 95% CI** | **Upper 95% CI** | **p-value** |
| **GC-MS peak area** | B-A | -0.0014 | -0.0063 | 0.0036 | 0.75 | 0.017 | -0.051 | 0.085 | 0.79 |
|  | C-A | -0.0013 | -0.0037 | 0.0062 | 0.77 | -0.20 | -0.26 | -0.13 | **<0.0001** |
|  | C-B | -0.0026 | -0.0023 | 0.0076 | 0.36 | -0.21 | -0.28 | -0.14 | **<0.0001** |
| **NMR peak area** | B-A | -0.0077 | -0.20 | 0.19 | 0.99 | -0.017 | -0.048 | 0.015 | 0.36 |
|  | C-A | -0.061 | -0.26 | 0.13 | 0.69 | -0.078 | -0.11 | -0.047 | **<0.0001** |
|  | C-B | -0.053 | -0.25 | 0.14 | 0.75 | -0.061 | -0.093 | -0.030 | **0.0005** |

^Bold values indicate p<0.05.^

**Table S5** GC-MS compounds listed in the Fig. 2**c** and Supporting Information Fig. S1**b** heat maps.

| **Compound** | | | |
| --- | --- | --- | --- |
| 1 | 10-hydroxydecanoic acid | 39 | D-ribose |
| 2 | uracil | 40 | D-saccharic acid |
| 3 | urea | 41 | D-xylose |
| 4 | 1-hexadecanol | 42 | erythritol |
| 5 | 1-methylhydantoin | 43 | fumaric acid |
| 6 | 2-hydroxybutyric acid | 44 | galactitol |
| 7 | 2-hydroxyglutaric acid | 45 | glyceric acid |
| 8 | 2-hydroxypyridine | 46 | glycerol 3-phosphate |
| 9 | 3-aminopropionitrile | 47 | glycolic acid |
| 10 | 3-hydroxy-3-methylglutaric acid (dicrotalic acid) | 48 | heptadecanoic acid |
| 11 | 3-hydroxybutyric acid | 49 | lactic acid |
| 12 | 4-guanidinobutyric acid | 50 | lauric acid |
| 13 | 4-hydroxy-3-methoxybenzoic acid (isovanillic acid) | 51 | L-glutamic acid |
| 14 | 4-hydroxybenzoic acid (p-salicylic acid) | 52 | L-isoleucine |
| 15 | acetyl-L-serine | 53 | L-proline |
| 16 | adipic acid | 54 | L-pyroglutamic acid |
| 17 | allo-inositol | 55 | L-serine |
| 18 | arabitol | 56 | L-threonine |
| 19 | arbutin | 57 | L-valine |
| 20 | benzene-1,2,4-triol | 58 | malonic acid |
| 21 | benzoic acid | 59 | myo-inositol |
| 22 | Beta- alanine | 60 | myristic acid |
| 23 | capric acid | 61 | oxalic acid |
| 24 | caprylic acid | 62 | palmitic acid |
| 25 | carbonate ion | 63 | Phosphate Ion |
| 26 | citraconic acid | 64 | phthalic acid |
| 27 | citramalic acid | 65 | pimelic acid |
| 28 | citric acid | 66 | porphine |
| 29 | D-arabinose | 67 | pyruvic acid |
| 30 | D-fructose | 68 | quinic acid |
| 31 | D-galactose | 69 | ribitol |
| 32 | D-gluconic acid | 70 | sedoheptulose anhydride monohydrate |
| 33 | D-glucose | 71 | stearic acid |
| 34 | DL-3-aminoisobutyric acid | 72 | succinic acid |
| 35 | D-lyxose | 73 | sucrose |
| 36 | D-malic acid | 74 | tagatose |
| 37 | D-mannitol | 75 | trans-aconitic acid |
| 38 | D-mannose |  |  |

**Table S6** GC-MS-identified compounds whose z-scores reflected the drought severity treatment gradient and increased or decreased from control to mild to severe drought severity (treatments A to B to C) before (T1) and after treatment (T2).

| **T1** | | **T2** | |
| --- | --- | --- | --- |
| **18% of compounds increased in T1 from A to B to C (38 of 206, 12 identified)** | **8% of compounds decreased in T1 from A to B to C (16 of 206, 3 identified)** | **38% of compounds increased in T2 from A to B to C (68 of 206, 28 identified)** | **6% of compounds decreased in T2 from A to B to C (12 of 206, 5 identified)** |
| 1-hexadecanol | Arbutin | 10-hydroxydecanoic acid | Adipic acid |
| 1-methylhydantoin | Succinic acid | 2-hydroxypyridine | Benzoic acid |
| Oxalic acid | 2-hydroxypyridine | 4-hydroxybenzoic acid (p-salicylic acid) | L-serine |
| Malonic acid |  | Citramalic acid | D-galactose |
| Allo-inositol |  | D-arabinose | Citraconic acid |
| Acetyl-L-serine |  | D-gluconic acid |  |
| Benzoic acid |  | D-lyxose |  |
| Heptadecanoic acid |  | D-**malic acid** |  |
| 2-hydroxyglutaric acid |  | D-mannitol |  |
| D-galactose |  | D-mannose |  |
| Arabitol |  | D-ribose |  |
| Pimelic acid |  | D-xylose |  |
|  |  | Erythritol |  |
|  |  | **Fumaric acid** |  |
|  |  | Galactitol |  |
|  |  | Glyceric acid |  |
|  |  | Lactic acid |  |
|  |  | L-isoleucine |  |
|  |  | L-threonine |  |
|  |  | Myo-inositol |  |
|  |  | Myristic acid |  |
|  |  | Palmitic acid |  |
|  |  | Phthalic acid |  |
|  |  | Pyruvic acid |  |
|  |  | Ribitol |  |
|  |  | Stearic acid |  |
|  |  | **Sucrose** |  |
|  |  | Tagatose |  |

^75 metabolites of the 206 total were identified by GC-MS. Bolded compounds were identified by both GC-MS and NMR (Table 3). 44% of GC-MS detected metabolites (i.e. 68+12=80 of 206) increased or decreased in T2 from A to B to C, while in T1 only 26% of GC-MS detected metabolites (i.e. 38+16=54 of 206) showed this pattern.^

**Table S7** NMR-identified compounds whose z-scores reflected the drought severity treatment gradient and increased or decreased from A to B to C before (T1) and after treatment (T2).

| **T1** | | **T2** | |
| --- | --- | --- | --- |
| **38% of compounds increased in T1 from A to B to C (5 of 13)** | **0% of compounds decreased in T1 from A to B to C (0 of 13)** | **38% of compounds increased in T2 from A to B to C (5 of 13)** | **31% of compounds decreased in T2 from A to B to C (4 of 13)** |
| Capric acid |  | **Sucrose** | Valeric acid |
| Butyric acid |  | **Malic acid** | 3-hydroxybutyric acid |
| Formic acid |  | Glucose | Butyric acid |
| Acetic acid |  | Betaine | Capric acid |
| Valeric acid |  | **Fumaric acid** |  |

^13 NMR metabolites were identified. Bolded compounds were identified by both GC-MS (Table 2) and NMR. 69% of NMR-identified metabolites (i.e. 5+4=9 of 13) increased or decreased in T2 from A to B to C, while in T1 only 38% of NMR identified metabolites (i.e. 5+0=5 of 13) showed this pattern.^


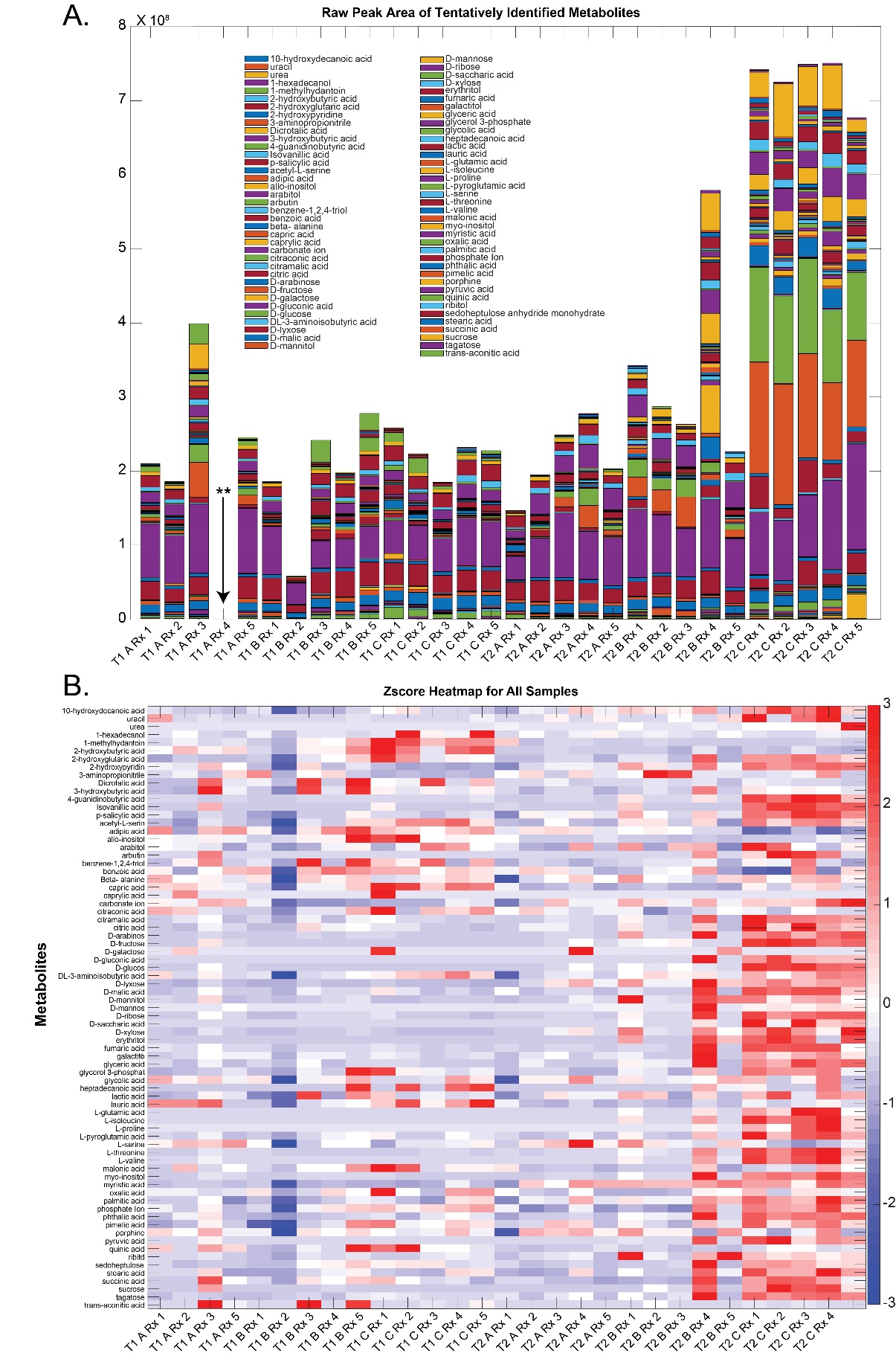


**Figure S1** GC-MS median of the raw peak areas for identified metabolites of individual samples (**a**). GC-MS heat map of z-scores for individual samples and identified metabolites (**b**).

*
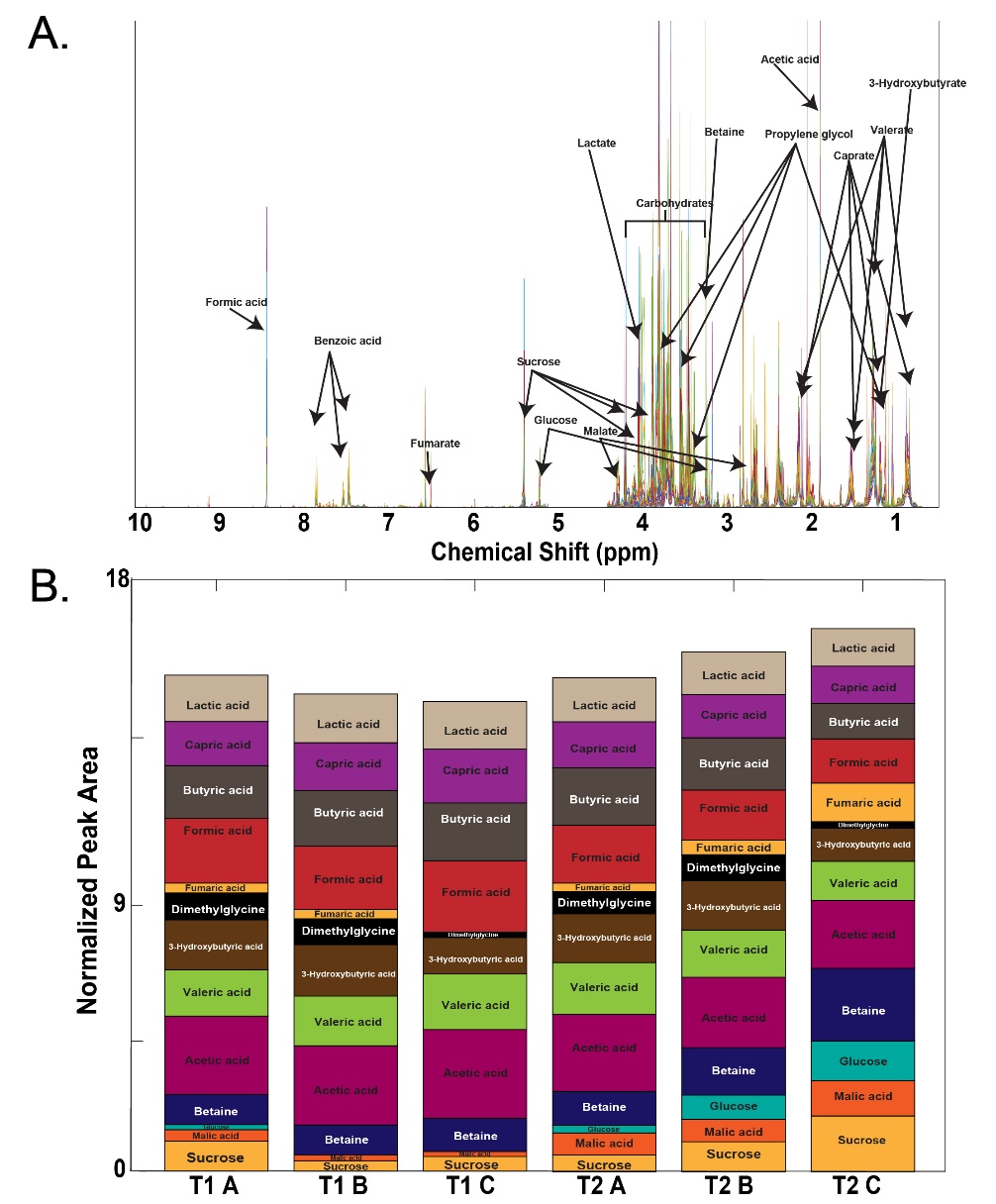
*

|  | **Metabolite** | **^1^H Chemical Shift (ppm)** | **^13^ C Chemical Shift (ppm)** |
| --- | --- | --- | --- |
| 1 | Sucrose | 5.41, 4.21, 4.19, 3.81, 3.66, 3.46 | 65.13, 64.10, 62.82 |
| 2 | Malate | 4.29, 2.68, 2.65, 2.37, 2.35, 2.33 |  |
| 3 | Glucose | 5.22, 4.63, 3.40, 3.38, 3.23 |  |
| 4 | Betaine | 3.25 |  |
| 5 | Acetic Acid | 1.90 | 26.01 |
| 6 | Valeric acid | 1.29, 0.88, 2.16, 1.52 | 15.86, 24.68, 30.33, 40.23 |
| 7 | 3-Hydroxybutyric acid | 4.31, 2.40, 2.29, 1.19 |  |
| 8 | Dimethylglycine | 2.93 |  |
| 9 | Fumaric acid | 6.51 |  |
| 10 | Formic acid | 8.44 |  |
| 11 | Butyric acid | 2.14, 1.55, 0.88 | 42.33, 22.02, 15.99 |
| 12 | Caprate or Caprylate | 0.85, 1.28, 1.54, 2.16 |  |
| 13 | Lactate | 1.32, 4.11 |  |

**Figure S2** All NMR spectra overlaid (**a**). Mean normalized peak area for the thirteen identified metabolites at T1 and T2 and treatment group (A, B, C) (**b**). Table lists NMR-identified metabolites and chemical shifts.


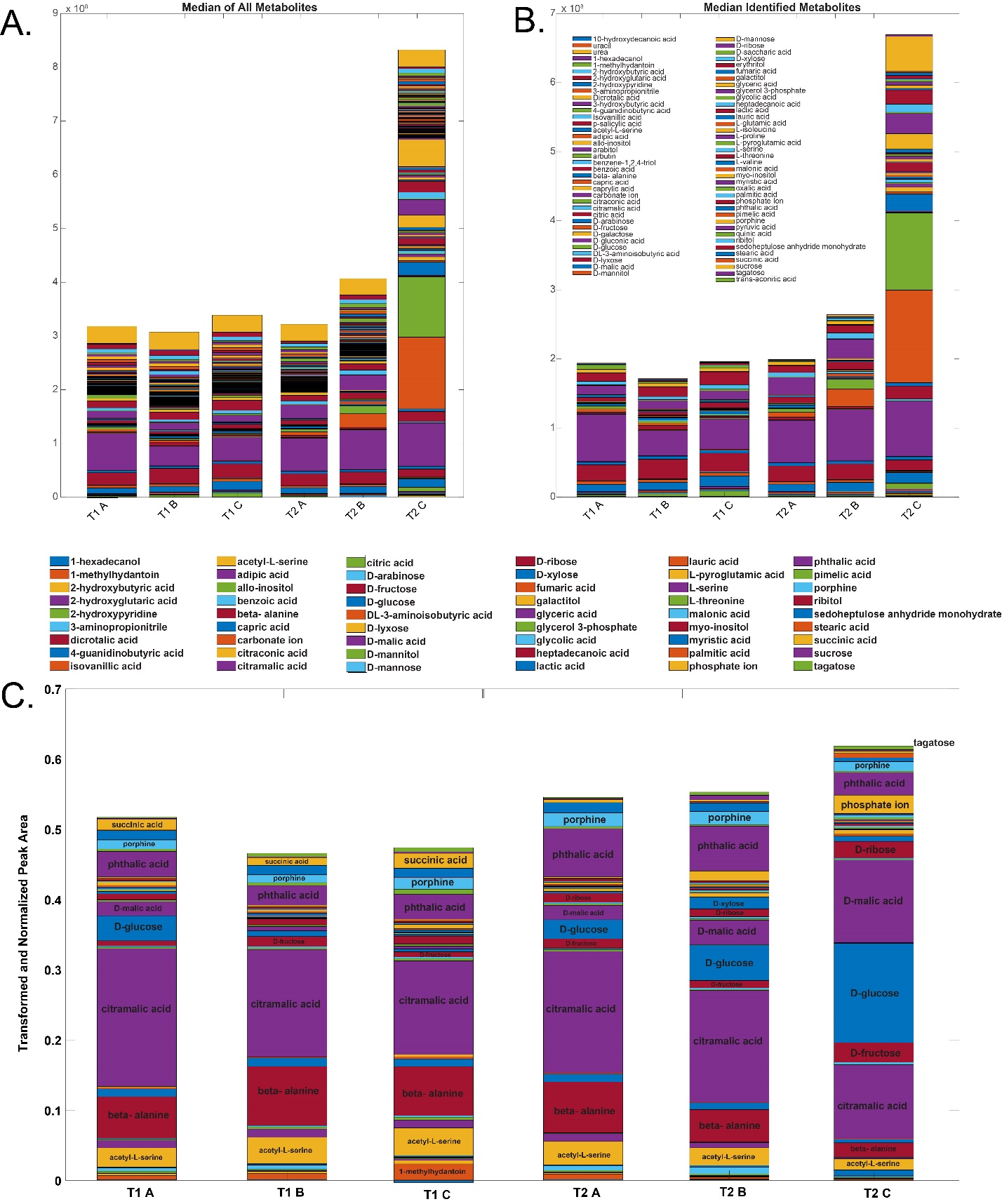


**Figure S3** GC-MS median of the raw peak areas for all metabolites (**a**) and only identified metabolites (**b**). GC-MS bar plot of transformed and normalized peak areas of identified metabolites that significantly differed between treatments (**c**).





**Figure S4** GC-MS probabilistic principal component analysis (pPCA) of identified metabolites only (**a**) of all treatments at T1 and T2. The top 20 metabolites driving each component are listed (**b**, **c**).





**Figure S5** GC-MS probabilistic principal component analysis (pPCA) on all identified and unidentified features (**a**) of all treatments at T1 and T2. The top ten metabolites driving the positive and negative pPCA loadings of each component are listed (**b**, **c**).





**Figure S6** NMR probabilistic principal component analysis (pPCA) on identified metabolites (**a**) of all treatments at T1 and T2. The top metabolites driving the positive and negative pPCA loadings of each component are listed (**b**, **c**).


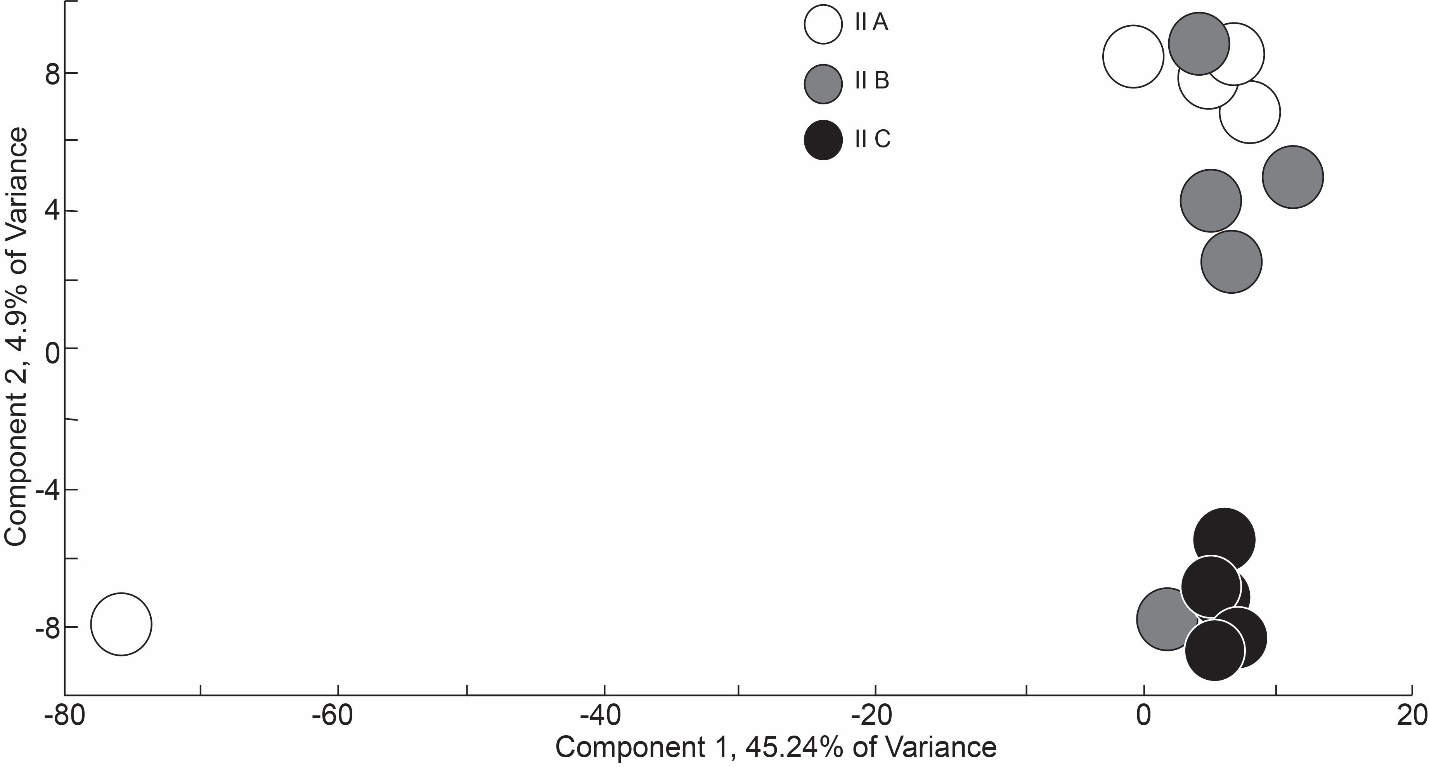


| **Top metabolites driving the positive loadings of component 2** | **Top metabolites driving the negative loadings of component 2** |
| --- | --- |
| Butyric acid | Sucrose |
|  | Betaine |
|  | Fumaric acid |

**Figure S7** NMR probabilistic principal component analysis (pPCA) of all treatments at T2 only. Table lists the top metabolites driving the positive and negative pPCA loadings of component 2.


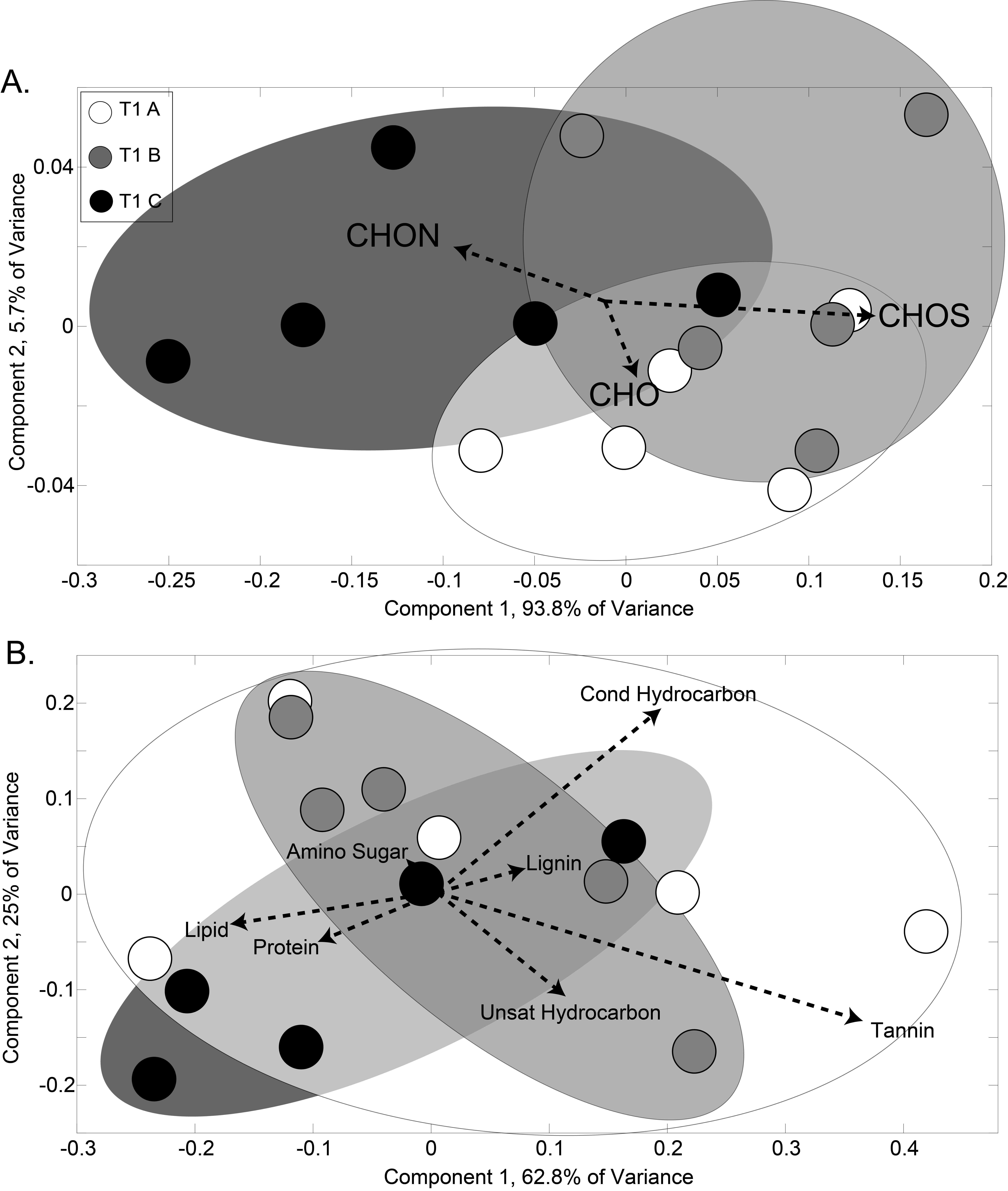


**Figure S8** FTICR principal components analysis (PCA) plots comparing treatments A, B, and C at T1 by elemental composition (**a**) and compound class (**b**). Angles between the vectors that are less than 90⁰ represent positive correlation and above 90⁰ represent negative correlation.

**Figure S9** Venn diagram of all (identified and unidentified) GC-MS metabolites found at T2 (including compounds also detected at T1).


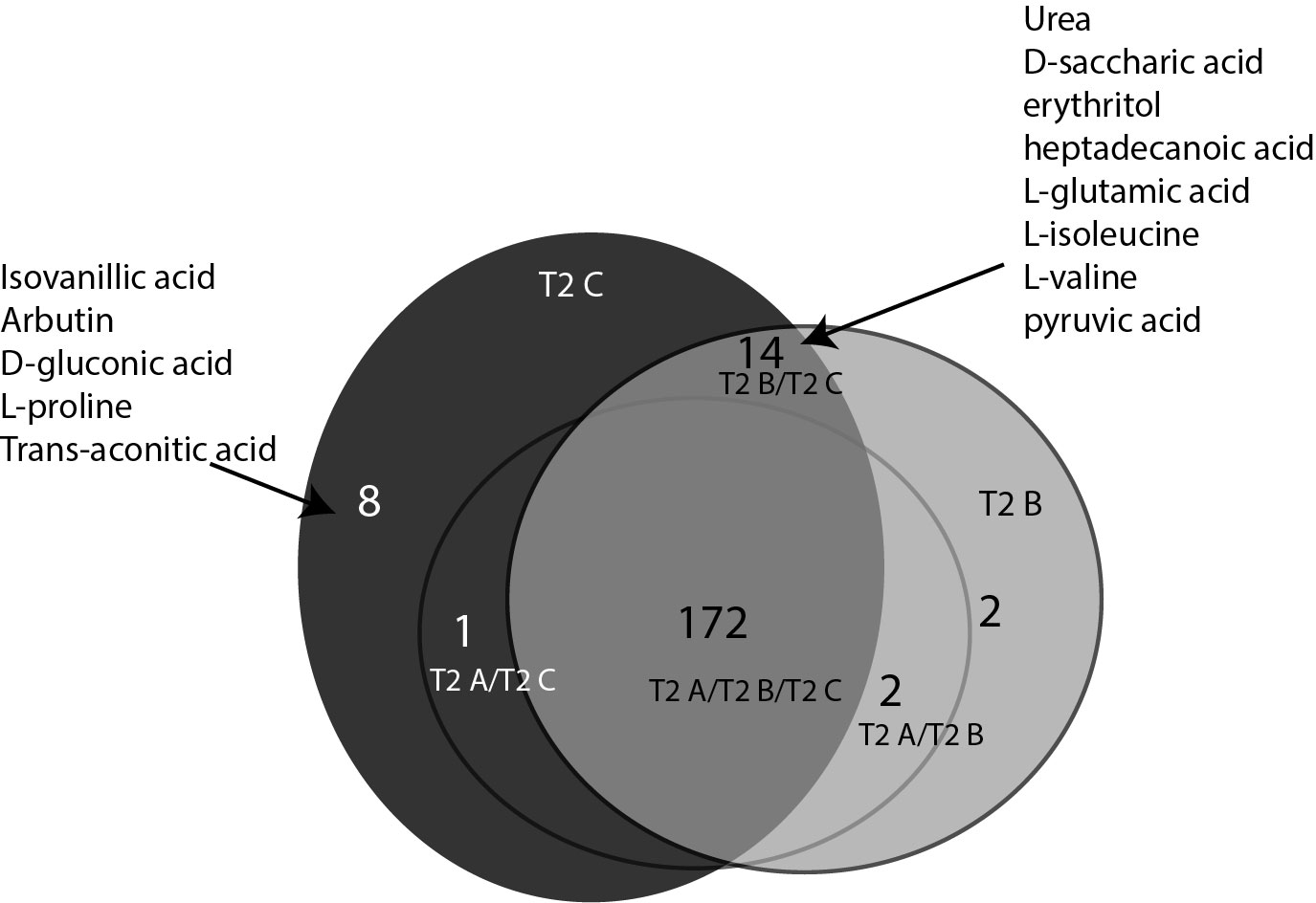


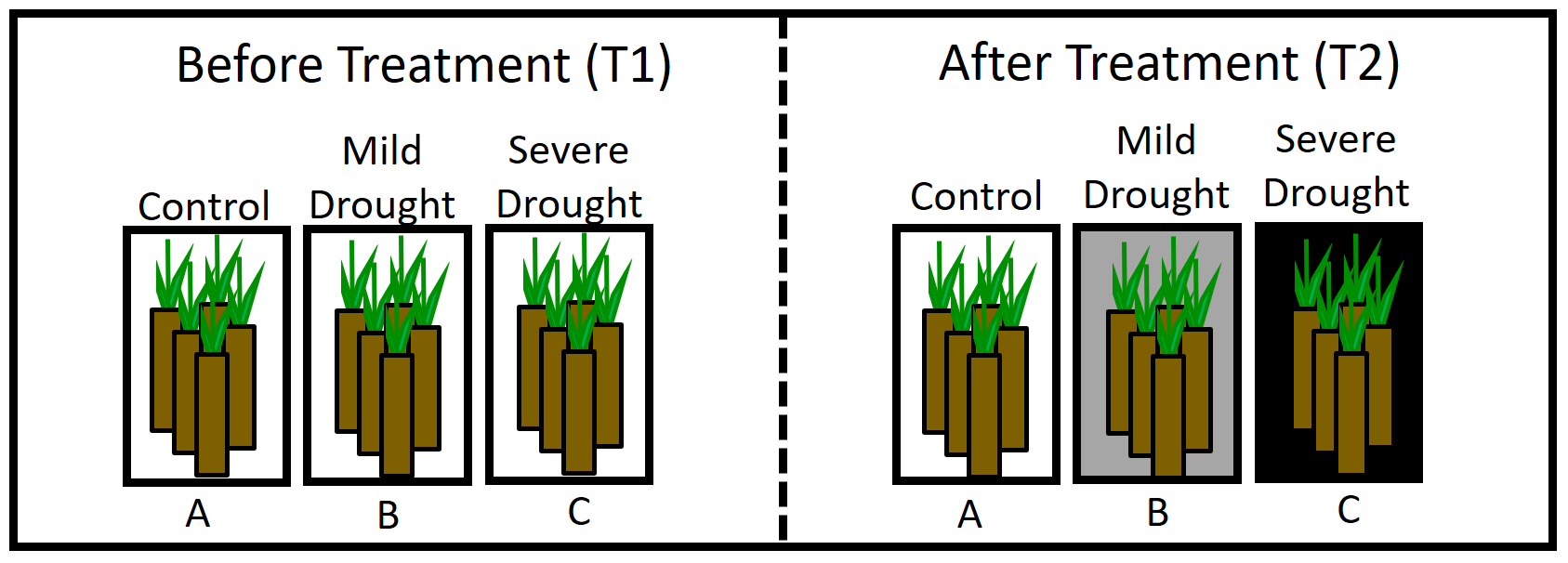


**Figure S10** The experiment consisted of three drought severity treatments (control = A, mild = B, severe = C) with measurements made before (T1) and after (T2) treatment. The same five individuals per treatment were sampled for predawn leaf water potential, gas exchange, biomass, and root exudates.
